# The efficacy of psychotherapy, pharmacotherapy and their combination on functioning and quality of life in depression: a meta-analysis

**DOI:** 10.1017/S0033291716002774

**Published:** 2016-10-26

**Authors:** K. Kamenov, C. Twomey, M. Cabello, A. M. Prina, J. L. Ayuso-Mateos

**Affiliations:** 1 Instituto de Salud Carlos III, Centro de Investigación Biomédica en Red, CIBERSAM, Madrid, Spain; 2 Department of Psychiatry, UniversityAutónoma de Madrid, Madrid, Spain; 3 Faculty of Social and Human Sciences, University of Southampton, Southampton, UK; 4 Health Service and Population Research Department, Centre for Global Mental Health, Institute of Psychiatry, Psychology and Neuroscience, King's College London, London, UK; 5 Instituto de investigación de la Princesa, (IIS-IP), Hospital Universitario de la Princesa, Madrid, Spain

**Keywords:** Depression, functioning, meta-analysis, pharmacotherapy, psychotherapy, quality of life

## Abstract

**Background:**

There is growing recognition of the importance of both functioning and quality of life (QoL) outcomes in the treatment of depressive disorders, but the meta-analytic evidence is scarce. The objective of this meta-analysis of randomized controlled trials (RCTs) was to determine the absolute and relative effects of psychotherapy, pharmacotherapy and their combination on functioning and QoL in patients with depression.

**Method:**

One hundred and fifty-three outcome trials involving 29 879 participants with depressive disorders were identified through database searches in Pubmed, PsycINFO and the Cochrane Central Register of Controlled Trials.

**Results:**

Compared to control conditions, psychotherapy and pharmacotherapy yielded small to moderate effect sizes for functioning and QoL, ranging from *g* = 0.31 to *g* = 0.43. When compared directly, initial analysis yielded no evidence that one of them was superior. After adjusting for publication bias, psychotherapy was more efficacious than pharmacotherapy (*g* = 0.21) for QoL. The combination of psychotherapy and medication performed significantly better for both outcomes compared to each treatment alone yielding small effect sizes (*g* = 0.32 to *g* = 0.39). Both interventions improved depression symptom severity more than functioning and QoL.

**Conclusion:**

Despite the small number of comparative trials for some of the analyses, this study reveals that combined treatment is superior, but psychotherapy and pharmacotherapy alone are also efficacious for improving functioning and QoL. The overall relatively modest effects suggest that future tailoring of therapies could be warranted to better meet the needs of individuals with functioning and QoL problems.

## Introduction

A considerable number of meta-analyses published in the last decade have clearly shown that both psychological and pharmacological treatments are efficacious for reducing symptoms in depression (Cuijpers *et al.* 2011, 2013; Spielmans *et al.* 2011). Recent literature, however, has suggested that functioning and quality of life (QoL) improvement might be equally important for people with depression as their symptom amelioration (Zimmerman *et al.* 2006; IsHak *et al.* 2011*a*; Lam *et al.* 2015). The Canadian Network for Mood and Anxiety Treatments (CANMAT) highlighted the need for evidence-based interventions that demonstrate improvement in functioning (Lam *et al.* 2015). From a clinical perspective, patients have prioritized functional over symptomatic outcomes and determined the return to a normal level of functioning at work, home or school as a significant factor for remission in depression (Zimmerman *et al.* 2006). Furthermore, improvement in QoL has been considered the ultimate outcome measure that indicates whether certain treatments have succeeded (IsHak *et al.* 2011*a*).

Despite the importance given to functioning and QoL, both dimensions remain under-researched in interventional studies (Kamenov *et al.* 2015). The terms have been used interchangeably in previous studies, but there is agreement that these concepts are not identical (Lam *et al.* 2015). Generally, functioning refers to one's performance in daily or social activities and QoL as one's satisfaction with these activities and perception of his/her health (IsHak *et al.* 2002, 2011*a*).

The conclusions drawn from the few published meta-analyses on functioning are limited. A review by De Silva *et al.* (2013) assessed the effect of psychosocial interventions on social functioning in depression. The article, however, reported only data from low- and middle-income countries. A later meta-analysis by Renner *et al.* (2014) also assessed the effect of psychotherapy on social functioning. The study, however, examined only the absolute efficacy of psychological interventions and certain functional difficulties such as problems in daily activities were not considered in the assessment of functioning. On the other hand, many meta-analyses have included QoL as a secondary measure of efficacy of various interventions (von Wolff *et al.* 2012; Spielmans *et al.* 2013). However, research so far has been mainly fragmentary, focusing only on specific types of treatments, and there exists only one narrative systematic review analyzing the impact of pharmacotherapy and psychotherapy on QoL in depression (IsHak *et al.* 2011*b*).

To our knowledge, there is no meta-analysis that comprehensively assesses the efficacy of interventions primarily aimed at depression treatment on functioning and QoL in depression. Determining this efficacy would have important implications for clinical decisions and policy making in terms of provision of treatments in primary and secondary mental health services. Therefore, this meta-analysis of randomized controlled trials aimed to assess (1) the effects of psychotherapy and pharmacotherapy compared to control conditions on functioning and QoL; (2) the effect of both when compared directly, and (3) the effect of their combination against either one. Additional sensitivity, subgroup and meta-regression analyses were performed.

## Method

Methods and results are presented according to the PRISMA statement for reporting systematic reviews (Moher *et al.* 2010).

### Search strategy

A systematic literature search combining the terms depressive disorder OR depression OR major depressive disorder (Mesh terms) AND functioning OR disability OR disability evaluation OR disabled persons OR sick leave OR activities of daily living OR leisure activities OR quality of life AND treatment OR intervention OR clinical trial OR therapy (MeSH terms, key words and text words) was performed in Pubmed, PsycINFO and the Cochrane Central Register of Controlled Trials. In the first two databases, the relevant option was selected to limit the search to Randomized Controlled Trials (the full search string can be seen in Supplementary material 3). Although non-randomized controlled trials provide valuable information in terms of ecological validity, RCTs minimize the influence of errors and bias on findings and offer the most rigorous method of determining whether a cause–effect relationship exists between treatment and outcome (Sibbald & Roland, 1998; Spring, 2007). Their sole inclusion safeguarded the validity of the findings and ensured methodological consistency. The search was performed in June 2015. The search was restricted by language (only articles published in English were considered) and age (only participants aged >18 years). In addition, the references of published meta-analyses and relevant articles were also checked.

### Study selection

The review included all randomized controlled trials that compared (1) psychotherapy or pharmacotherapy against treatment as usual (TAU), placebo, waiting list (WL) or other control group: (2) psychotherapy against pharmacotherapy; or (3) the combination of psychotherapy and pharmacotherapy against either one. Psychotherapy was defined by the American Psychiatric Association as ‘the informed and intentional application of clinical methods and interpersonal stances derived from established psychological principles for the purpose of assisting people to modify their behaviors, cognitions, emotions, and/or other personal characteristics in directions that the participants deem desirable’ (Norcross, 1990). More specifically, different psychotherapeutic approaches were defined according to definition previously developed in comparative meta-analyses (Cuijpers *et al.* 2008*a*). All studies had to report at least one validated outcome measure assessing functioning (any difficulty experienced in maintaining daily activities or participation in social life (Lam *et al.* 2015) or QoL (one's satisfaction with these activities and perception of his/her health (World Health Organization Quality of Life Group, 1997; IsHak *et al.* 2002). Information on symptom severity was extracted only from validated instruments that explicitly measured symptoms of depression [e.g. Hamilton Depression Rating Scale (HAMD; Hamilton, 1960)]. The diagnosis of depression had to be established by a standardized diagnostic interview according to ICD or DSM criteria (APA, 1980, 1987, 2000; WHO, 1992). Studies including bipolar or schizoaffective disorder or reporting results from maintenance or continuation therapies were excluded. The abstract screening was done by one researcher (K.K.) and a random selection of 20% of the abstracts was double-checked independently by another two researchers (M.C. and C.T.).

### Data extraction and quality assessment

Data from the selected studies were extracted by one researcher (K.K) and checked for consistency independently by two other researchers. Divergences were resolved by consensus. In case of missing data, authors were contacted. When results from more than one outcome measure assessing the same concept (either functioning or QoL) were available in a study, data from all were extracted and combined as a mean effect size. To avoid double counting, the effects of different intervention arms representing the same generic intervention (e.g. GP-delivered psychotherapy and clinician-delivered psychotherapy) included in a single study were averaged and entered once in the analysis (Senn, 2009). SF-36 (Ware & Sherbourne, 1992) was considered as an outcome measure of QoL(IsHak *et al.* 2011*b*) but if a study reported post assessment score on the social functioning subdomain, it was included separately as an outcome measure of functioning. Global measures of functioning were considered only if they included domains of social functioning and daily activities (De Silva *et al.* 2013). Data on effect estimates were extracted at post-assessment. The instruments were patient self-assessments and clinician-rated tools.

Four criteria of the Cochrane Collaboration risk of bias tool were used for assessing methodological quality of the studies – sequence generation, allocation concealment, blinding of assessors, and incomplete outcome data (Higgins *et al.* 2011). It is impossible for the majority of psychotherapeutic designs to employ a double blind design, therefore blinding of assessors in these studies was adapted to include only outcome assessors in masking procedures.

### Statistical analyses

Statistical analyses were performed using the program Comprehensive Meta-Analysis, version 2.0 (www.meta-analysis.com/). The effect size for each individual meta-analysis was calculated, aggregating the pooled difference between the two groups of treatments at the end of the intervention. Hedges' *g* was preferred as an effect estimate because of its capability to provide a better effect estimate for small sample sizes (Deeks *et al.* 2008). The magnitude of the effect size may be interpreted as small (0.2), medium (0.5), and large (0.8) (Cohen, 1988). We used a random effects meta-analysis model which assumes that variance in observed effects is explained not only by sampling variability (as in fixed effect analysis) but also real differences in treatment effects resulting from heterogeneity in study populations, intervention delivery, follow-up length and other factors (Riley *et al.* 2011). To test the heterogeneity, Higgins' *I*^2^ statistic was calculated. A value of 0% indicates no heterogeneity, 25% indicate low heterogeneity, 50% – moderate heterogeneity, and 75% high heterogeneity (Higgins *et al.* 2003). Publication bias was assessed in each of the meta-analyses by visual inspection of the funnel plots and the trim-and-fill procedure to analyze the changes after the accounting for publication bias (Duval & Tweedie, 2000). In addition to the analyses on functioning and QoL, we performed a series of individual meta-analyses to assess the effect of psychotherapy, pharmacotherapy and their combination on depression symptom severity. The outcome was a reduction of symptom severity according to the instruments' scores.

In order to check the robustness of the results, sensitivity analyses were conducted. First, the main analyses were repeated after exclusion of low-quality studies. Then, to test whether one single outcome measure had a strong impact on the overall effect size, a series of sensitivity analyses were performed after the exclusion of each of the instruments. Lastly, the effect size was calculated for studies with a treatment duration of ⩽3 months and compared with studies with a treatment duration of >3 months. The results of the sensitivity analyses were considered ‘consistent’ with the primary analysis if there was no change in the magnitude of the effect size (from high to moderate, from moderate to small, etc.). Since the selected studies were heterogeneous with respect to comparator groups, study populations, included interventions and outcome measures, series of subgroup analyses were performed. We examined whether there were differences in terms of age groups – adults (18–65 years) *v.* older adults (>65 years), psychotherapies [Cognitive Behavioral Therapy (CBT), Interpersonal Therapy (IPT), Problem Solving Therapy (PST), others], medication [selective serotonin reuptake inhibitors (SSRIs), serotonin-norepinephrine reuptake inhibitors (SNRIs), tricyclic antidepressants (TCAs), others], control groups (WL, TAU, Placebo, others), outcome measures, duration of treatment (3 months *v.* >3 months) and types of depression (major depressive disorder, dysthymia, subthreshold depression, others).Long-term effects were not assessed, because a very small number of studies reported any follow-up data and the reported outcomes differed widely between studies. Follow-up periods differed significantly (e.g. 3 months *v.* 12 months) and the nature of the follow-ups was different: some studies reported only naturalistic outcomes, whereas others delivered booster sessions and maintenance treatments during the follow-up period. A mixed-effects model, combining a random-effects model within subgroups and a fixed-effects model across subgroups, was used. Multivariate meta-regression analyses were conducted using Stata v. 12.0 for Windows (Stata Corporation, USA). In these analyses the outcome variable was the weighted effect sizes of psychotherapy, pharmacotherapy or their combination on functioning and QoL at post treatment. The predictors were severity of depression (effect size at post treatment), number of psychotherapeutic sessions (where possible), duration of treatment in weeks, duration of trial in weeks, and year of publication. All the predictors used were continuous variables. The regression coefficient obtained from the meta-regression analysis revealed how the intervention effect changes with a unit increase in the predictors and whether there was a linear relationship between the intervention effect and the predictors.

## Results

### Study selection

**Fig. 1.**
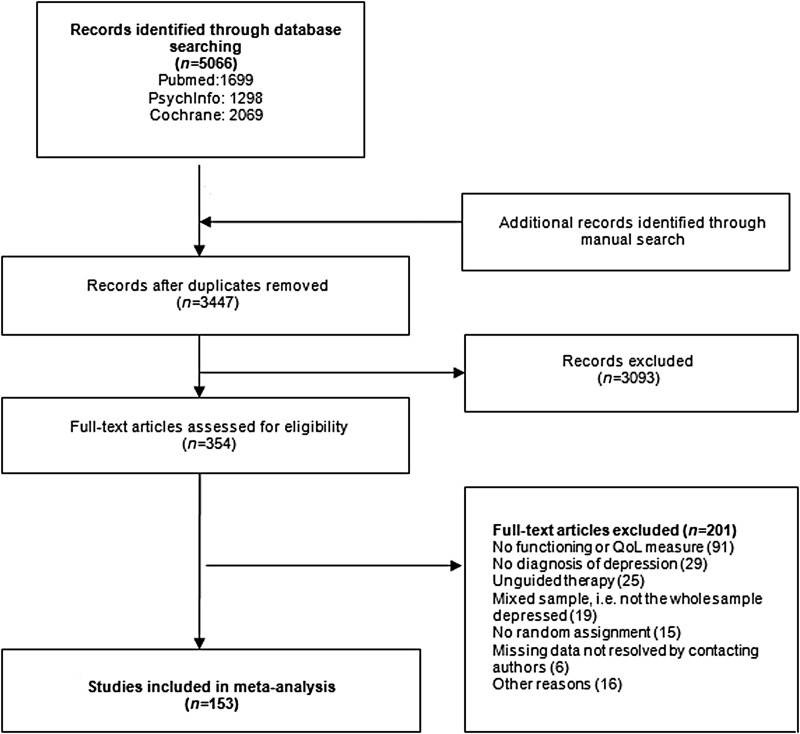
Flow chart of study selection.

After removal of duplicates, 3447 articles were identified for abstract check. Of these, 354 were selected for full-text screening. 153 articles met the inclusion criteria and were included in the analyses. The main reasons for exclusion were lack of functional or QoL measures and non-standardized diagnosis of depression. Some studies included outcome measures for both functioning and QoL, resulting in their inclusion in more than one analysis. The selection process can be seen in Fig. 1.

### Description of included studies

Selected characteristics of the studies can be seen in Table 1. A total of 29 879 participants were included in all trials. The majority of the participants were adults aged between 18 and 65 years, and 60.1% of all individuals had major depressive disorder. The duration of the trials ranged from 4 weeks to 1 year. The most common psychotherapeutic intervention found in the literature was CBT, based on two specific tasks – cognitive restructuring and behavioral approach (such as exposure and response prevention). Interpersonal therapy – a structured therapy with a predominant focus on addressing interpersonal issues – was also commonly used in studies. The number and format of psychotherapeutic sessions differed across studies, ranging between 4 and 20, weekly and bi-weekly, individual and group sessions. We defined pharmacotherapy as any treatment by means of pharmaceutical drugs, e.g. antidepressants. The most frequently used drug in the studies was duloxetine. The dosage given to participants varied depending on the type of drug and the duration of the trials.

**Table 1.** Selected characteristics of the included studies (N = 153)

Characteristic	*N* (studies)	%
Diagnosis		
Major depressive disorder	92	60.1
Dysthymia	22	14.4
Subthreshold depression	10	6.5
Other	29	18.9
Target group		
Adults	120	78.4
Older adults	15	9.8
Women	18	11.8
Type of psychotherapy		
Cognitive Behavioral Therapy	31	31.3
Interpersonal Therapy	17	17.2
Problem Solving Therapy	9	9.1
Other	42	42.4
Type of pharmacotherapy		
Selective serotonin reuptake inhibitors	35	37.6
Selective serotonin and norepinephrine reuptake inhibitors	31	33.3
Tricyclic antidepressants	19	20.4
Other	8	8.6
Study quality		
⩽2	75	49
⩾3	78	51
Country		
USA	71	46.4
UK	28	18.3
Netherlands	11	7.2
Others	45	29.4

In terms of instruments for measuring functioning, the Sheehan Disability Scale (SDS; Sheehan, 1983) and Social Adjustment Scale (SAS; Weissman *et al.* 1978) were the most commonly used ones, and for QoL – the Quality of Life Enjoyment and Satisfaction Questionnaire (Q-LES-Q; Endicott *et al.* 1993) and SF-36. The majority of the trials were conducted in USA, UK or The Netherlands. The quality of the studies varied. There were 47 trials (30.7%) meeting all four quality criteria, whereas 75 studies (49%) were missing two or more components. A full table including all study characteristics and references of the included articles can be found in Supplementary material 1.

### Psychotherapy and pharmacotherapy *v.* control condition

**Fig. 2.**
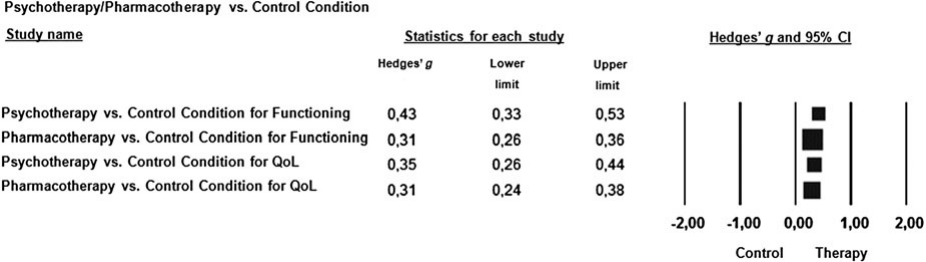
Total standardized effect sizes (Hedges' *g*) of psychotherapy and pharmacotherapy against control condition for functioning and QoL.

Fig. 2 provides information on the total effects of each of the four individual meta-analyses (full details on individual studies are available in Supplementary material 2A and 2B). Compared to control conditions, both psychotherapy and pharmacotherapy had small to moderate effects on functioning, with slight superiority of psychotherapy. The mean effect of psychotherapy on functioning resulting from 52 comparisons was *g* = 0.43 [95% confidence interval (CI) 0.33–0.54; *I*^2^ = 74.94, 95% CI 67.24–80.27]. After adjusting for publication bias, the effect size decreased to 0.35 (95% CI 0.24–0.46). For pharmacotherapy, the 53 comparisons yielded an effect of *g* = 0.31 (95% CI 0.26–0.36; *I*^2^ = 64.91, 95% CI 51.66–73.21). After adjusting for publication bias, the effect size decreased to 0.27 (95% CI 0.21–0.32).

For QoL, both psychotherapy and pharmacotherapy yielded small effect sizes. The 37 comparisons yielded a mean effect of psychotherapy (*g* = 0.35, 95% CI 0.26–0.44; *I*^2^ = 68.24, 95% CI 53.74–76.65). The effect of pharmacotherapy coming from 33 studies was *g* = 0.31 (95% CI 0.24–0.38; *I*^2^ = 81.18, 95% CI 74.25–85.55).

### Psychotherapy *v.* pharmacotherapy

**Fig. 3.**
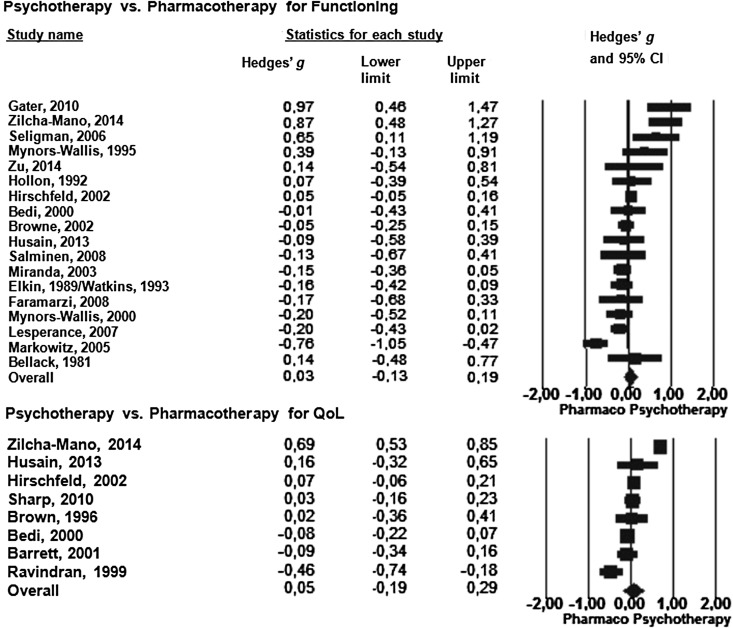
Standardized effect sizes (Hedges' *g*) of psychotherapy against pharmacotherapy on functioning and QoL.

For both functioning and QoL, there was no significant difference between therapies. In terms of functioning, the mean effect size was 0.03 (95% CI −0.13 to 0.19; *I*^2^ = 77.85, 95% CI 63.98–84.79) in favor of psychotherapy (Fig. 3). After adjusting for publication bias, Hedges' *g* was still insignificant, but increased substantially to 0.12 (95% CI −0.06 to 0.30) in favor of psychotherapy. For QoL, the effect size was 0.05 (95% CI −0.19 to 0.29; *I*^2^ = 90.72, 95% CI 84.47–93.71) in favor of psychotherapy. After adjusting for publication bias, the effect size was small, but significant in favor of psychotherapy (*g* = 0.21, 95% CI 0.01–0.43).

### Combination of psychotherapy and pharmacotherapy *v.* either one

**Fig. 4.**
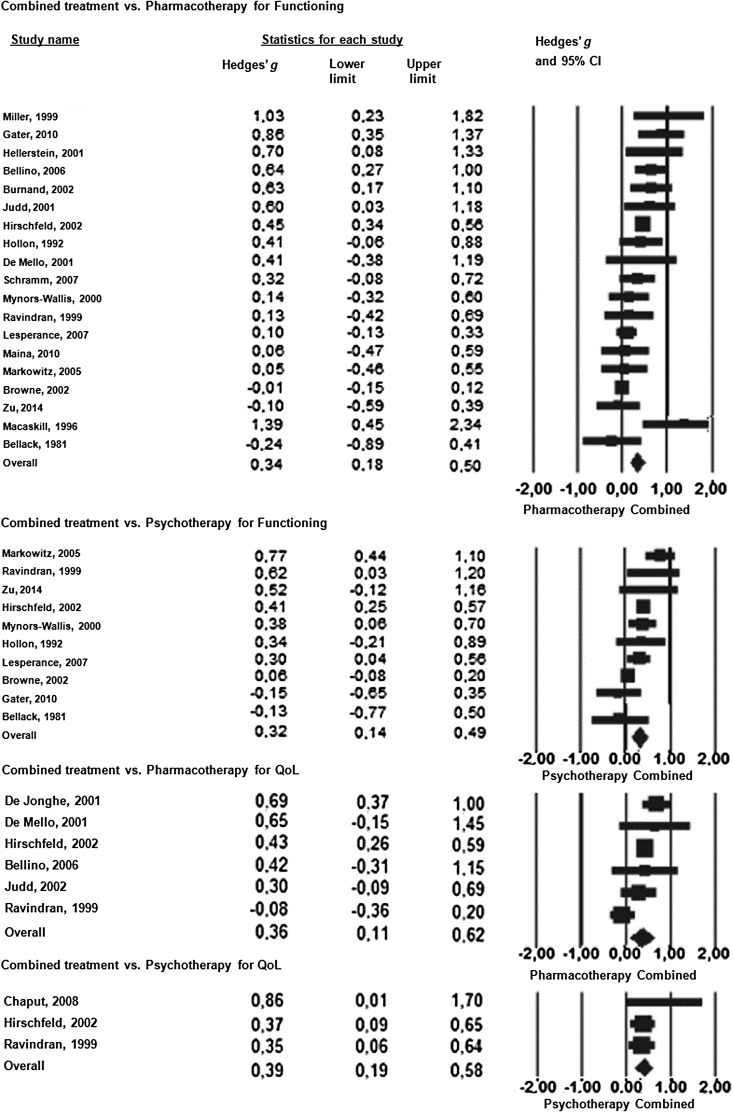
Standardized effect sizes (Hedges' *g*) of combined treatment against psychotherapy and medication alone on functioning and QoL.

The effects of the direct comparisons between combination of psychotherapy and pharmacotherapy against either one on functioning or QoL are presented in Fig. 4. In all four analyses, the combined treatment was significantly superior to each treatment alone yielding small effect sizes. For functioning, the 19 comparisons between combined treatment and pharmacotherapy alone resulted in effect size of *g* = 0.34 (95% CI 0.18–0.50; *I*^2^ = 69.51, 95% CI 47.22–79.85) in favor of combined treatment. When combined treatment was compared to psychotherapy alone in 10 studies, the analysis yielded an effect size of 0.32 (95% CI 0.14–0.49; *I*^2^ = 66.98, 95% CI 21.02–81.43).

Six studies compared combined treatment against pharmacotherapy and three against psychotherapy on QoL. This weakened the power of the analysis. Compared to medication, combined treatment was significantly more efficacious (*g* = 0.36, 95% CI 0.11–0.62; *I*^2^ = 66.91, 95% CI 0.00–84.11). The studies comparing combined treatment with psychological interventions yielded an effect size of 0.39 (95% CI 0.19–0.58) in favor of combined treatment.

### Effect of psychotherapy and pharmacotherapy on depressive symptoms

Psychotherapy showed a better result (*g* = 0.60, 95% CI 0.51–0.68; *I*^2^ = 80.15, 95% CI 75.53–83.52) than pharmacotherapy (*g* = 0.33, 95% CI 0.29–0.38; *I*^2^ = 54.37, 95% CI 35.77–65.71) when both interventions were compared to control condition. After adjusting for publication bias, the effect of psychotherapy dropped to *g* = 0.45, whereas the effect of pharmacotherapy remained similar (*g* = 0.30). When both treatments were compared directly, there was no statistically significant difference (*g* = −0.03, 95% CI −0.15 to 0.10) in favor of medication. The combination of treatments was superior to psychotherapy and pharmacotherapy alone, yielding small effect sizes, *g* = 0.30 (95% CI 0.16–0.45) and *g* = 0.34 (95% CI 0.18–050), respectively.

### Sensitivity and subgroup analyses

The sensitivity analyses revealed some major differences in the effects of psychotherapy, pharmacotherapy and their combination on functioning and QoL according to the duration of the interventions applied. When psychotherapy was compared directly to medication on QoL, the trials with ⩽3 months treatment duration yielded an effect of *g* = −0.08 (95% CI −0.26 to 0.09) in favor of medication, whereas trials with a treatment duration of >3 months showed superiority of psychotherapy (*g* = 0.26, 95% CI −0.24 to 0.76). The same applied for combined treatment against pharmacotherapy for QoL (*g* = 0.22, 95% CI −0.12 to 0.56 *v. g* = 0.64, 95% CI 0.37–0.92, respectively). The subsequent subgroup analyses comparing the duration of treatment, however, found no significant differences among studies. This might be due to low power because of the low number of studies included in the analyses.

Furthermore, sensitivity analyses were performed after the exclusion of low quality studies. For all analyses we found small deviations of the effect sizes, which did not affect the magnitude of the effect estimates. However, subgroup analyses were conducted to compare high quality (meeting three or four components of the Cochrane risk of bias tool) to low quality (missing two or more components). Results revealed significant changes only in studies comparing pharmacotherapy to control conditions on functioning (high-quality studies: *g* = 0.26, 95% CI 0.21–0.31 *v.* low quality: *g* = 0.36, 95% CI 0.28–0.44, *p* = 0.05) and QoL (high quality: *g* = 0.22, 95% CI 0.11–0.33 *v.* low quality: *g* = 0.36, 95% CI 0.27–0.45 *p* < 0.05). Last, to investigate the impact of individual outcome measures on the overall effect sizes, we conducted a series of sensitivity analyses. Here, we excluded one instrument at a time and examined consequent deviations in effect sizes. For all analyses, we found small deviations of the effect sizes of no more than 0.10, which indicated that no individual outcome measure had a strong impact on the overall effect size. The subsequent subgroup analyses comparing grouped studies according to the instruments used did not show any significant differences across subgroups.

Differences in the effects of psychotherapy compared to TAU, WL or placebo on functioning and QoL were also assessed in subgroup analyses. The effect of psychotherapy on functioning was significantly higher (*p* < 0.05) in studies with waiting list controls (*g* = 0.61, 95% CI 0.40–0.81) than in studies with TAU (*g* = 0.36, 95% CI 0.24–0.48). The effect size of studies comparing psychotherapy to waiting list (*g* = 0.47, 95% CI 0.34–0.59) on QoL was significantly higher (*p* < 0.05) than studies with TAU (*g* = 0.34, 95% CI 0.23–0.45) or placebo controls (*g* = 0.20, 95% CI 0.03–0.37). Similar subgroup analyses could not be performed for pharmacotherapy, as 95% of the studies used placebo controls. Furthermore, clinician-rated scales were compared to self-rated tools. Studies applying clinician-rated tools yielded slightly higher effect sizes in all analyses performed, but statistically significant differences were not found. Regarding age groups, only studies comparing pharmacotherapy to control condition for QoL revealed significant difference between age groups (*g* = 0.35, 95% CI 0.27–0.42 for adults *v. g* = 0.16, 95% CI 0.04–0.27 for older adults). The rest of the subgroup analyses did not reveal any significant differences across subgroups for depression type (major depressive disorder, dysthymia, subthreshold depression, others), type of psychotherapy – CBT, IPT, PST, or others, or type of medication – SSRIs, SNRIs, TCAs, or others. All subgroups were directly compared to each other, or each subgroup was compared to the other subgroups pooled. All subgroup analyses are available upon request.

### Meta-regression analyses

Multivariate meta-regression analyses assessing potential predictors were performed. The effect size of depression severity was a significant predictor of the effects of psychotherapy and pharmacotherapy on functioning (*B* = 0.59, 95% CI 0.42–0.76, *p* < 0.001 and *B* = 0.94, 95% CI 0.59–1.29, *p* < 0.001, respectively) and QoL (*B* = 0.35, 95% CI 0.1–0.61, *p* < 0.01 and *B* = 0.94, 95% CI 0.59–1.30, *p* < 0.001) when they were compared to control conditions, and when pharmacotherapy was compared directly to psychotherapy (*B* = 29.55, 95% CI 5.83–53.27, *p* < 0.05) and combined treatment (*B* = 0.001, 95% CI 0.0004–0.002, *p* < 0.01) for functioning. This indicates when symptom severity is reduced, the effect size of psychotherapy and pharmacotherapy on improving functioning and QoL increases. The remaining predictors – number of sessions, duration of treatment, and duration of trial – were not significant in any of the meta-regression analyses we performed. Number of sessions (*B* = 0.02, 95% CI 0.09–0.60, *p* < 0.05) and year of publication (*B* = 0.001, 95% CI 0.0003–0.002, *p* < 0.01) were found significant only when the effect of psychotherapy on QoL was compared to control conditions. This indicated that the effect size of psychotherapy on QoL increases with higher number of psychotherapeutic sessions and in recent publications. All analyses can be found in Supplementary material 4.

## Discussion

This meta-analysis was the first to systematically assess the effects of psychotherapy, pharmacotherapy and their combination on improvements in functioning and QoL in depressive disorders. The study demonstrates that the combination between psychotherapy and pharmacotherapy perform significantly better than each intervention alone for both outcomes. Psychotherapy and pharmacotherapy alone are also efficacious for improving functioning and QoL, although showing only small to moderate effects. When compared directly, in initial analysis there was no significant difference between the interventions. After adjusting for publication bias psychotherapy was more efficacious than pharmacotherapy for QoL.

Our results are consistent with the two previously published meta-analyses on psychotherapy for social functioning. Both Renner *et al.* (2014) and De Silva *et al.* (2013) found effect sizes of *g* = 0.46 in favor of psychotherapy over control condition, which was similar to the result obtained in this study – 0.43. Even though psychotherapy showed slightly superior absolute effects to medication on both functioning and QoL, it has to be noted that the great majority of included pharmacological studies involved random assignment to a blinded control condition as opposed to the psychological trials, comparing interventions to WL or TAU control groups. It has been argued that awareness of treatment assignment might produce expectancy effects in the intervention group and despair in the control group, leading to inflated effect sizes in favor of psychotherapy. On the other hand, assignment to a blinded condition controls for expectancy effects and induction of hope, thus suggesting eventual underestimation of the effects of medication compared to psychotherapy (Gaudiano & Herbert, 2005). Nonetheless, a recent meta-analysis by Cuijpers *et al.* (2015) comparing pharmacological studies involving or missing double blind condition to psychotherapy did not find any difference in the effects of both groups.

We compared the effect of both interventions on functioning and QoL but no significant differences were found. This is consistent with previous meta-analytic evidence on depressive symptoms, where no superiority was found for any of the intervention types (Cuijpers *et al.* 2013). Still, when studies were adjusted for publication bias, psychotherapy was slightly better for improving functioning (*g* = 0.12) and statistically superior than pharmacotherapy on QoL (*g* = 0.21). These results, although suggesting the slight superiority of psychological over antidepressant treatment for functioning and QoL, are not robust enough to suggest priority when clinical or policy decisions are made. There is no clear economic evidence that psychotherapy should be a preferable treatment choice compared to pharmacotherapy (Bosmans *et al.* 2008). However, a recent meta-analysis reveals a strong patient preference for psychological treatment over medication (McHugh *et al.* 2013). Moreover, evidence states that the majority of people expressing personal preference for psychological therapy choose not to get treated at all rather than receive medication (Layard *et al.* 2007). Alongside the benefits of pharmacotherapy for depression, it is also worth taking into account that potential side-effects and adverse events related to the use of medication may have a detrimental impact on functioning and QoL. A review by Kelly *et al.* (2008) showed that people with depression experience diminished QoL related to troublesome side effects. Further research is needed to investigate the role of side effects in the efficacy of interventions for depression. Even though the number of studies directly comparing psychotherapy and pharmacotherapy was not very high, our results warrant future research to determine the economic costs and benefits of eventual enhanced provision of psychotherapeutic treatment.

The subgroup analyses found higher effect estimates for psychotherapy against waiting list compared to TAU and placebo for functioning and QoL. This finding was somewhat expected and consistent with previous meta-analyses for depression (Cuijpers *et al.* 2008*b*). Waiting list control conditions involve no actual treatment and thus positive outcomes for psychotherapy are relatively easy to attain. Comparison to treatment as usual is more demanding, because it involves usual care provided in healthcare settings and the effect estimate shows the true additional benefit of psychotherapy on the outcome. Although not to a significant level, we found that studies applying clinician-rated scales yielded slightly higher effect sizes than studies that relied on self-rated tools. The absence of significance may be partly explained by an absence of power– only a small number of studies used clinician-rated tools. Tentatively, this trend is in line with the results of previous psychotherapy meta-analyses indicating that clinician-rated instruments are associated with higher effect-sizes of functioning and depressive symptom severity (Cuijpers *et al.* 2010; Renner *et al.* 2014). In the absence of a gold standard measure for functioning (Lam *et al.* 2015; Madden *et al.* 2015), inclusion of both types of outcome measures may be warranted to facilitate comprehensive assessments in future meta-analyses.

Psychotherapy and pharmacotherapy showed higher effect sizes on reducing depressive symptoms although there was a strong indication for publication bias. When the effects of psychotherapy and pharmacotherapy on depressive symptoms were compared to control conditions, psychotherapy showed better results (*g* = 0.60 *v. g* = 0.33, respectively). This result, however, has to be considered with caution, because in studies directly comparing both interventions, we did not find a significant difference between the interventions (*g* = −0.03). Moreover, a previous meta-analysis (Cuijpers *et al.* 2013) found no superiority of one intervention over another. Future meta-analyses of comparative outcome studies should shed more light on potential differences in efficacy between psychotherapeutic and pharmacological treatments. Such investigations should also take into account patient preferences and costs.

The results from the meta-regression analysis suggest that functioning and QoL improve when symptom severity improves, but which is the leading factor is still unknown. Previous research suggests that functional recovery appears later than the symptomatic one and certain level of impairment continues even after the symptomatology is ameliorated, and that depressive symptoms and QoL do not share high proportion of common variance (Coryell *et al.* 1993; Trompenaars *et al.* 2006). The residual functional impairment has been found to evoke relapse and recurrences (Vittengl *et al.* 2009); therefore functioning and QoL should be directly targeted in the response and remission criteria for a more comprehensive assessment of treatment efficacy. There are already steps in this direction. Individual Burden of Illness Index for depression was created to measure treatment impact and recovery in depression by incorporating symptom severity, functioning, and QoL outcomes (Cohen *et al.* 2013). Zimmerman *et al.* (2014) validated the Remission from Depression Questionnaire, including different domains of functioning and QoL along with symptomatology. However, all attempts for implementation of such criteria are still in their infancy and future research is warranted.

The present meta-analysis demonstrates that the combination of psychotherapy and pharmacotherapy is significantly better than any of the treatments alone for both functioning and QoL. The number of studies comparing treatments for QoL was limited, but still our result has an important clinical implication for primary and secondary mental health professionals when choosing their treatment lines. Recent data showing the trends in treatment of depression report decrease in the use of combined treatment and psychotherapy and a substantial increase in the prescription of antidepressants (Gemmill *et al.* 2008; Marcus & Olfson, 2010). This might be driven by various factors such as availability of resources in terms of money and personnel. However, a recent analysis by Sado *et al.* (2009) shows that combined therapy for depression appears to be cost-effective from health-care system and social perspective. More cost effectiveness and comparative long-term data on combined treatment is needed (McAllister-Williams, 2006).

This study has to be seen in light of certain limitations. First, half of the included trials had low quality. This questions the robustness of the results. However, the sensitivity and subgroup analysis we performed did not reveal significant differences in the effects between high and low quality studies. Second, for some of the individual analyses the number of studies was not large enough to allow for generalizability of results. Furthermore, mainly overall improvements in functioning and QoL were assessed. There was a lack of domain-specific reporting that could have provided information on the effects of interventions on specific areas of functioning and QoL. This meta-analysis was based on study-level data. Individual patient level meta-analysis based on original datasets of the included studies could have revealed differences among first cases of depression and recurrent depression, level of severity, or allowed better analysis of predictors of depression. A further limitation was our inability to analyze long-term outcomes and their interactions, due to the lack of follow-up data. Follow-up data would allow for investigating long-term effects of interventions and temporal relationships between changes in functioning, QoL and severity of symptoms. Future longitudinal epidemiological studies could fill this research gap and provide important information on the course of functioning in depression. Last, only articles in English were considered. This might have omitted relevant information.

In conclusion, this meta-analysis provides comprehensive evidence that existing psychological and pharmacological interventions are efficacious for improving functioning and QoL in depression. There is no robust evidence that one of the interventions is superior, although psychotherapy appears slightly superior to medication. The combination between psychotherapy and medication performs significantly better for both outcomes when compared to each treatment alone. The relatively modest effects suggest that future research should focus on tailoring therapies to better cover the needs of individuals, implementation of instruments assessing both outcomes as primary outcome measures in trials, and reporting domain-specific changes across treatments for better understanding of the course of depression.

## Supplementary material

For supplementary material accompanying this paper visit http://dx.doi.org/10.1017/S0033291716002774. click here to view supplementary material
