# Supplementary material for: The efficacy of psychotherapy, pharmacotherapy and their combination on functioning and quality of life in depression: a meta-analysis
Source: Psychol Med. 2016 Oct 26;47(3):414–25. doi: 10.1017/S0033291716002774 (PMC5244449; doi:10.1017/S0033291716002774)
Supplement: Supplementary file 1 [file S0033291716002774sup001.zip › Supplementary material 1.docx]

| **Study** | **Definition** | **Target Group** | **Duration of treatment in weeks and follow-up**  **(if available)** | **Therapy (format)** | **N** | **Session/Dosage mg/day** | **Control** | **N** | **Outcome Measure Functioning** | **Outcome Measure**  **QoL** | **Outcome Measure Depression** | **Quality (1-4)** | **Country** |
| --- | --- | --- | --- | --- | --- | --- | --- | --- | --- | --- | --- | --- | --- |
| Agosti, 1991  Alegria, 2014 | MDD/Dys  Depression | Adults  Adults | 6  8 (2 monthfollow-up) | Phenelzine, imipramine, l-deprenyl  ECLA-T/ECLA-F | 38  84 | 60-90mg for phen/200-300mg for imipr/40mg for l-deprenyl  8 | placebo  TAU | 23  86 | LIFE (psychosocial functioning section)  WHODAS | -  - | -  PHQ-9/HSCL-20 | 1  2 | USA  USA |
| Allart-Van Dam, 2003 | Subthr.  Depression | Adults | 12 | CWD (group) | 61 | 12 | TAU | 41 | SIG | - | BDI | 3 | Netherlands |
| Andersson, 2005 | MDD | Adults | 10 (6 monthfollow-up) | ISH, based on CBT | 36 | 5 | Web-based discussion | 49 | - | QOLI | BDI/MADRS | 4 | Sweden |
| Araya et al., 2003 | MDD | Women | 12 (6 monthfollow-up) | Psychoeducation | 104 | 7 | TAU | 109 | SF-36 Social Functioning Scale | SF-36 | HAMD | 4 | Chile |
| Asnis et al., 2013 | MDD | Adults | 8 | Levomilnacipran | 176 | 40, 80, 120mg* | placebo | 175 | SDS | - | MADRS/HAMD | 4 | USA |
| Bakish, 2014 | MDD | Adults | 10 | Levomilnacipran | 185 | 40,80mg | placebo | 185 | SDS | - | MADRS | 4 | Canada/USA |
| Barge-Schaapveld, 2002 | MDD | Adults | 6 | Imipramine | 32 | 50-200mg** | placebo | 31 | - | mQOL/QOL-Vas | HAMD | 1 | Netherlands |
| Barrett, 2001 | Dys/Min D | Adults | 11 | PST (individual)/paroxetine | 80/80 | 6/10-40mg | Placebo | 81 | - | SF-36 | HCSL/HAMD | 4 | USA |
| Bass, 2006 | MDD | Adults | 16 (6 monthfollow-up) | IPT (group) | 103 | 16 | TAU | 113 | Other |  | HSCL | 4 | Uganda |
| Bedi, 2000 | MDD | Adults | 8 | Counseling | 40 | 6 | AT | 45 | SF-36 social functioning Scale | SF-36 | BDI | 2 | UK |
| Bellack, 1981 | Depression | Women | 12 | Social Skills/amitriptyline | 20/18 | 12/50-300mg | Combined treatment | 17 | SAS-SR | - | HAMD/BDI | 1 | USA |
| Bellino, 2006 | MDD | Adults | 24 | IPT (individual) | 16 | 24/20-40mg | fluoxetine / Comb | 16/ 16 | IIP-64/SAT-P | - | HAMD | 1 | Italy |
| Berger, 2011 | MDD | Adults | 10 (6 monthfollow-up) | GSH | 25 | 10 | WL | 26 | IIP-64 | WHOQOL-BREF | BDI-II | 4 | Switzerland |
| Blum, 2015 | MDD | Adults | 8 | Levomilnacipran | 529 | 40, 80, 120mg | placebo | 175 | SF-36 Social functioning Scale | SF-36 | MADRS/HAMD | 4 | USA |
| Bowie, 2013 | MDD | Adults | 10 | CR | 17 | 10 | WL | 16 | LIFE-RIFT | - | MADRS | 4 | Canada |
| Boyer, 2008 | MDD | Adults | 8 | Desvenlafaxine | 163/157 | 50, 100mg | placebo | 159 | SDS | - | HAMD/MADRS | 2 | Canada |
| Brown, 1996 | MDD | Adults | 8 | IPT (individual) | 29 | 16 | nortriptyline | 21 | - | SF-36 | HAMD | 3 | USA |
| Browne, 2002 | Dys | Adults | 24 (18 monthfollow-up) | IPT (individual) | 178 | 10/50-200mg | Sertraline/ Comb | 196/212 | SAS-SR/FAD | - | MADRS/CES-D | 4 | Canada |
| Burke, 2002 | MDE | Adults | 8 | Escitalopram | 118/123 | 10,20mg | placebo | 125 | - | Quality of Life Questionnaire | MADRS/HAMD | 2 | USA |
| Burnand, 2002 | MDD | Adults | 10 | PP (individual)/ Clomipramine | 35 | 25-125mg | Clomipramine | 39/35 | GAS | - | HAMD | 3 | Switzerland |
| Burt, 2005 | MDD | Women | 9 | duloxetine | 55 | 60mg | placebo | 59 | - | QLDS | HAMD | 2 | USA |
| Carlbring, 2013 | MDE | Adults | 8 (3 monthfollow-up) | iBA with ACT | 40 | 7 | WL | 40 | - | QOLI | MADRS/BDI | 2 | Sweden |
| Carpenter, 2002 | MDE | Adults | 4 | Mirtazapine | 11 | 15-30mg | placebo | 15 | GAF | Q-LES-Q | HAMD/IDS-SR | 1 | USA |
| Carta, 2012 | Depression | Adults | 24 | CBC | 34 | 12 | TAU | 30 | - | WHOQOL-BREF | BDI | 1 | Italy |
| Casanas, 2012 | MDD | Adults | 12 (3 and 6 monthfollow-up) | Psychoeducation | 119 | 12 | TAU | 112 | - | EQ-5D | BDI | 3 | Spain |
| Chaput, 2008 | MDD | Adults | 12 | CBT (individual) + quetiapine | 11 | 12/ 25mg | CBT + placebo | 11 | - | Q-LES-Q | MADRS | 4 | Canada |
| Choi, 2011 | MDD | Adults | 8 | GSH: CBT | 25 | 6 | WL | 30 | SDS | - | BDI | 2 | Australia |
| Clarke, 2005 | Depression | Adults | 16 | iBasedCR Mail Reminder/Phone reminder | 75/80 | 7 | TAU | 100 | - | SF-12 | CES-D | 2 | USA |
| Clayton, 2013 | MDD | Women | 8 | Desvenlafaxine | 211 | 50mg | placebo | 211 | SDS | EQ-5D | HAMD/MADRS/QIDS-SR | 2 | USA |
| Coventry, 2015 | Depression | Adults | 16 | CC | 153/152 | 8 | TAU | 163/167 | SDS | WHOQOL-BREF | SCL | 4 | UK |
| Cramer, 2011 | Depression | Women | 12 (3 monthfollow-up) | Group CBT | 45 | 12 | TAU | 19 | - | SF-12 | PHQ-9 | 2 | UK |
| De Graaf, 2009 | Depression | Adults | 8 (1 and 4 monthfollow-up) | CCBT | 100 | 8 | TAU | 103 | WSAS | SF-36 | BDI-II | 2 | Netherlands |
| De Jonghe, 2001 | MDD | Adults | 24 | SPSP + medic. | 80 | 16 | AT | 81 | - | QLDS | HAMD/SCL | 3 | Netherlands |
| De Mello, 2001 | Dys | Adults | 32 (4 and 6 monthfollow-up) | IPT-D (individual)/Moclobemide | 11 | 16/150-300mg | Moclobemide | 13 | GAF | QOLE | BDI/MADRS | 2 | Brazil |
| Detke, 2002 | MDD | Adults | 9 | Duloxetine | 121 | 60mg | placebo | 115 | - | QLDS | HAMD | 2 | USA |
| Detke, 2002² | MDD | Adults | 9 | Duloxetine | 128 | 60mg | placebo | 139 | - | QLDS | HAMD | 2 | USA |
| Devanand, 2005 | Dys | Older Adults | 9 | Fuoxetine | 44 | 20-60mg | placebo | 46 | - | Q-LES-Q | HAMD/BDI | 4 | USA |
| Dobkin, 2011 | MDD/ Dys/D NOS | Adults | 10 (1 monthfollow-up) | CBT (individual) | 41 | 10 | TAU | 39 | SF-36 Social Functioning Scale | SF-36 | HAMD/BDI | 4 | USA |
| Dowrick, 2000 | Depression | Adults | 24 (6 and 12 monthfollow-up) | PST (individual)/CWD (group) | 98/80 | 6 for PST, 8 for CWT | TAU | 139 | SF-36 Social Functioning Scale | SF-36 | BDI | 4 | UK |
| Duarte, 2009 | MDD | Adults | 12 (6 monthfollow-up) | CBT (individual) | 41 | 12 | TAU | 44 | - | KDQOL-SF | BDI | 3 | Brazil |
| Dubini, 1997 | MDD | Adults | 8 | Reboxetine, fluoxetine | 103/100 | 8-10mg rebox/20-40mg fluox. | placebo | 99 | SASS | - |  | 2 | Italy/France |
| Dunlop, 2011 | MDD | Adults | 12 | Desvenlafaxine | 285 | 50mg | placebo | 142 | SDS | Q-LES-Q | HAMD | 2 | USA |
| Ekers, 2011 | Depression | Adults | 12 | BA (Individual) | 16 | 12 | TAU | 22 | WSAS | - | BDI-II | 4 | UK |
| Elkin, 1989 | MDD | Adults | 16 | CBT (individual)  IPT (individual)  Imipramine | 120/57 | 16 for CBT and IPT/285mg | placebo | 62 | GAS | - | HAMD/BDI | 4 | USA |
| Faramarzi, 2008 | MDD | Women | 12 | CBT (group)/Fluox | 29 | 10/10-20mg | TAU | 30 | GHQ social dysfunction | - | GHQ depression | 1 | Iran |
| Fledderus, 2012 | Other | Adults | 9 | GSH: ACT - E/ACT-M | 125/125 | 9 | WL | 126 | MHC-SF-SOC | - | CES-D | 2 | Netherlands |
| Furukawa, 2012 | Subthr. Depression | Adults | 16 | tCBT | 58 | 8 | WL | 60 | HPQ | - | BDI-II | 3 | Japan |
| Gater, 2010 | Depression | Women | 12 (6 monthfollow-up) | SI (individual) | 31 | 10 | AT/Comb | 35 /29 | SFQ | - | HAMD | 3 | UK |
| Gaynor, 2011 | MDD | Adults | 8 | Duloxetine | 250 | 60mg | placebo | 260 | SDS | - | MADRS | 2 | USA |
| Gaynor, 2011² | MDD | Adults | 8 | Duloxetine | 255 | 60mg | placebo | 265 | SDS | - | MADRS | 2 | USA |
| Gellis, 2008 | Min D | Older Adults | 6 (3 and 6 monthfollow-up) | PST (individual) | 30 | 6 | TAU | 32 | - | QOLI | GDS/HAMD | 3 | USA |
| Goldstein, 2004 | MDD | Adults | 8 | Duloxetine /paroxetine | 84/86/84 | Dul:40,80; Par: 20mg | placebo | 88 | - | QLDS | MADRS/HAMD | 2 | USA |
| Gommol, 2014 | MDD | Adults | 24 | Levomilnacipran | 174 | 40-120mg | placebo | 181 | SDS | SF-36 |  | 4 | USA |
| Gottlieb, 2007 | Depression | Adults | 12 | Paroxetine | 14 | 12.5-25mg | placebo | 14 | SF-36 Social Functioning Scale | SF-36 | BDI | 1 | USA |
| Grote, 2009 | PPD | Women | 24 | Brief IPT (individual) | 25 | 8 | TAU | 28 | SAS-SR | - | BDI | 2 | USA |
| Haringsma, 2006 | MDD | Older Adults | 10 | CWD (group) | 21 | 10 | WL | 22 | MOS-SF-20 social/role functioning | MOS-SF | CES-D/HADS-d | 2 | Netherlands |
| Harley, 2008 | MDD | Adults | 16 | DBT (group) | 10 | 16 | WL | 9 | SAS-SR/LIFE-RIFT | - | BDI/HAMD | 2 | USA |
| HDTSG, 2002 | MDD | Adults | 8 | Sertraline | 109 | 50mg | placebo | 116 | GAF | - | HAMD/BDI | 4 | USA |
| Healy, 2000 | MDE | Adults | 8 | Reboxetine/fluox | 103/100 |  | placebo | 99 | SASS | - | HAMD/MADRS | 1 | UK |
| Heiligenstein, 1995 | MDD | Older Adults | 6 | Fluoxetine | 261 | 20mg | placebo | 271 | SF-36 Social Functioning Scale | SF-36 |  | 2 | USA |
| Hellerstein, 2001 | Dys | Adults | 36 | CIGP-CD (group) | 20 | 16/10-80mg | Fluoxetine/Comb | 20 /20 | GAF | - | HAMD/BDI | 0 | USA |
| Hellerstein, 2010 | Dys | Adults | 12 | Escitalopram | 17 | 20mg | placebo | 19 | SAS/GAF | - | HAMD/BDI | 2 | USA |
| Hellerstein, 2012 | Dys/D NOS | Adults | 10 | Duloxetine | 29 | 30-120mg | placebo | 28 | SAS/GAF | - | HAMD/BDI | 3 | USA |
| Hewett, 2009 | MDD | Adults | 8 | Bupropion , Venlafaxine | 187/187 | Bu:150/Ven:75mg | placebo | 197 | SDS | Q-LES-Q | MADRS | 2 | UK |
| Hewett, 2010 | MDD | Older Adults | 10 | Bupropion | 188 | 150-300mg | placebo | 181 | SDS | - | MADRS | 2 | UK |
| Higgins, 2004 | Depression | Older Adults | 4 (2 monthfollow-up) | ConT | 10 | 4 | TAU | 9 | HSQ-12 | - | HAMD | 1 | UK |
| Hirschfeld, 2002 | MDD | Adults | 12 | CBASP (individual) | 227 | 20/200-300mg | Nefazodone/Comb | 225/226 | SAS | SF-36 | HAMD | 4 | USA |
| Hoifodt, 2013 | Depression | Adults | 6 (6 monthfollow-up) | Therapist-assisted Web-based CBT | 52 | 5 | WL | 54 | - | EQ-5D | BDI-II | 4 | Norway |
| Hollon, 1992 | MDD | Adults | 12 | CT/Imipramine | 25/57 | 20/75-300mg | Comb | 25 | GAS | - | HAMD/BDI | 2 | USA |
| Husain, 2013 | Depression | Women | 12 (3 monthfollow-up) | PI (group), based on CBT | 32 | 10 | fluoxetine | 32 | BDQ | EQ-5D | HAMD | 4 | UK/Pakistan |
| Ince, 2013 | Depression | Adults | 6 (4 monthfollow-up) | iPST | 38 | 5 | WL | 36 | - | EQ-VAS | CES-D | 2 | Netherlands |
| Iwata, 2013 | MDD | Adults | 8 | Desvenlafaxine | 232/236 | 25,50mg | placebo | 231 | SDS | - | HAMD/MADRS | 4 | USA/JAPAN |
| Johansson, 2012 | MDD | Adults | 10 | iCBTtailored vs. no tailored | 36/37 | 8 | Other | 42 | - | QOLI | BDI/MADRS | 3 | Sweden |
| Jonkers, 2012 | Min D/MDD/ Dys | Older Adults | 12 (6 monthfollow-up) | MPI | 125 | 10 | TAU | 133 | ADL/ IPA | - |  | 4 | Netherlands |
| Judd, 2001 | MDD | Adults | 12 | FEPP/Venlafaxine | 12 | 12/75-150mg | Venlafaxine | 19 | GAF, SF-36 social functioning scale | SF-36 | BDI | 1 | Australia |
| Judd, 2004 | Min D | Adults | 12 | Fluoxetine | 78 | 10,20mg | placebo | 79 | GAF, MOS-36 social functioning scale |  | IDS/HAMD/BDI | 2 | USA |
| Kanter, 2015 | MDD | Adults | 12 | BA | 21 | 12 | TAU | 22 | - | Q-LES-Q | HAMD/BDI-II | 3 | USA |
| Keitner, 2009 | MDD | Adults | 4 | Risperidone | 64 | 0.5-3mg | placebo | 30 | - | Q-LES-Q | HAMD/MADRS | 2 | USA |
| Kessler, 2009 | Depression | Adults | 16 (4 monthfollow-up) | iCBT | 99 | 10 | TAU | 91 | - | EQ-5D | BDI | 4 | UK |
| Kim, 2015 | MDD/Min D | Adults | 24 | Escitalopram | 108 | 5-20mg | placebo | 109 | WHODAS/ SOFAS | - | HAMD/BDI/MADRS | 2 | Korea |
| Klug, 2010 | MDD | Older Adults | 52 | GHT | 30 | 51 home visits | TAU | 30 | GAF | BELP–KF | GDS-15 | 4 | Austria |
| Kocsis, 1988 | Dys | Adults | 6 | imipramine | 22 | 50-300mg | placebo | 24 | SAS-SR | - | HAMD | 1 | USA |
| Kocsis, 1997 | Dys | Adults | 12 | Sertraline/Imipramine | 127/126 | 50-200/50-300mg | placebo | 130 | GAF, SAS | Q-LES-Q |  | 2 | USA |
| Korte, 2012 | MDE | Adults | 12 (3 monthfollow-up) | LRT | 100 | 8 | TAU | 102 | - | EQ-5D | CES-D | 3 | Netherlands |
| Laidlaw, 2008 | MDD | Older Adults | 8 (3 and 6 monthfollow-up) | CBT (individual) | 20 | 8 | TAU | 20 | WHOQOL Social Rel. Scale | WHOQOL | HAMD/BDI/GDS | 2 | UK |
| Lenderking, 1999 | MDD | Adults | 8 | Venlafaxine | 426 | 25-200mg | placebo | 174 | AQ | - | HAMD | 2 | USA |
| Lesperance, 2007 | MDD | Adults | 12 | IPT/citalopram/ Comb | 142/142 | 4/20mg | TAU/placebo/ | 142/142 | FPI/IPRI | - | HAMD/BDI-II/IDS | 4 | France |
| Liebowitz, 2008 | MDD | Adults | 8 | Desvenlafaxine 50 mg/day and 100 mg/day | 148/142 | 50,100mg | placebo | 149 | SDS | - | HAMD/MADRS | 3 | USA |
| Liebowitz, 2013 | MDD | Adults | 8 | Desvenlafaxine | 226/224 | 8 | placebo | 223 | SDS | - | HAMD/MADRS | 3 | USA |
| Locklear, 2013 | MDD | Adults | 11 | Quetiapine | 164 | 50-300mg | placebo | 171 | - | Q-LES-Q | MADRS | 2 | USA |
| Lydiard, 1997 | MDD | Adults | 8 | Sertraline, amitriptyline | 126/124 | 50-200mg sertr/50-150mg amitr. | placebo | 122 | GAS | - | HAMD-BDI | 2 | USA |
| Lynch, 1997 | MinD | Adults | 6 | PST (telephone) | 7 | 6 | TAU | 9 | DUKE social functioning  scale | - | HAMD | 1 | USA |
| Lynch, 2004 | Depression | Adults | 6 | tPST (individual) | 9 | 6 | TAU | 13 | DUKE social functioning scale | - | BDI | 2 | USA |
| Macaskill, 1996 | MDD | Adults | 24 | RET+lofepramine | 10 | 30/35-280mg | lofepramine | 10 | SAS-SR | - | HAMD/BDI | 0 | UK |
| Mahmoud, 2007 | MDD | Adults | 6 | Risperidone | 137 | 1-2mg | placebo | 131 | SDS | Q-LES-Q | HAMD | 4 | USA |
| Maina, 2010 | MDD | Adults | 52 | BDT/Medication | 25 | 10-16/flu: 100-300/ser: 50-200mg | Fluvoxamine or sertraline | 29 | GAF | - | HAMD | 3 | Italy |
| Markowitz, 2005 | Dys | Adults | 16 | IPT/BSP (individual) | 23/26 | 16-18 | Sertraline /Comb | 24 /21 | IIP-64/SAS | - | BDI/HAMD | 3 | USA |
| McIntyre, 2014 | MDD | Adults | 8 | Quetiapine | 61 | 50-150mg | placebo | 59 | SDS, GAS | Q-LES-Q | HAMD | 2 | Canada |
| Meeks, 2015 | MDD | Older Adults | 10 (3 and 6 monthfollow-up) | BAI (individual) | 23 | 10 | TAU | 26 | Dartmouth COOP Scales of Functioning - COOP daily activities and Social Functioning | - | GDS | 2 | USA |
| Miller, 1999 | MDD+Dys | Adults | 24 (6 monthfollow-up) | CBT + amitriptyline, desipramine | 14 | 20/150mg | Amitriptyline or desipramine | 12 | SAS-SR | - | HAMD/BDI | 3 | USA |
| Miller, 2002 | MDD/Dys/ D NOS | Women | 12 | tIPT (individual ) | 15 | 12 | TAU | 15 | SAS-SR | - | HAMD/BDI | 2 | USA |
| Miranda, 2003 | MDD | Women | 24 (1 and 4 monthfollow-up) | CBT (individual/group) | 90 | 8 | TAU | 89 | SAS-CR; SF-36 social functioning scale | - | HAMD | 4 | USA |
| Montgomery, 2013 | MDD | Adults | 10 | Levomilnacipran | 276 | 75-100mg | placebo | 277 | SDS | - | HAMD/MADRS | 4 | UK |
| Moritz, 2012 | Depression | Adults | 8 | Self Help iDeprexis | 80 | 10 | WL | 90 | - | WHOQOL-BREF | BDI | 3 | Germany |
| Mynors-Wallis, 1995 | MDD | Adults | 12 | PST, Amitriptyline | 29/27 | 6/50-150mg | Placebo | 26 | SAS-SR | - | HAMD/BDI | 4 | UK |
| Mynors-Wallis, 2000 | MDD | Adults | 12 (10 monthfollow-up) | PST (GP or nurse) | 39/41 | 6/flu:100;par:20mg | Fluvoxamin/Paroxetine/Comb | 36 /35 | SAS-SR | - | HAMD/BDI | 4 | UK |
| Naeem, 2014 | DE/RDE | Adults | 12 | CaCBT | 94 | 9 | TAU | 89 | BDQ | - | HADS depression | 4 | Pakistan |
| Naugebauer, 2006 | Depression | Women | 6 | IPC | 10 | 6 | TAU | 9 | SF-36 role functioning scale | - | HAMD | 3 | USA |
| Nickel, 2005 | MDD | Women | 10 | Topiramate | 32 | 50-200mg | placebo | 32 | SF-36 social functioning scale | SF-36 | HAMD | 2 | Germany |
| Oakes, 2012 | MDD | Adults | 8 (1 monthfollow-up) | Duloxetine | 194/195 | 60mg | placebo | 59/63 | SDS/SASS | - | HAMD | 4 | USA |
| O’Hara, 2000 | MDD | Women | 12 | IPT (individual) | 48 | 12 | WL | 51 | SAS-SR | - | BDI/HAMD | 3 | USA |
| Pangallo, 2011 | MDD | Adults | 10 | LY2216684 | 197 | 6-18mg | placebo | 217 | SDS | EQ-5D | MADRS | 4 | USA |
| Pedersen, 2002 | MDD | Adults | 6 | Venlafaxine/imipramine | 99/96 | 75-225mg | placebo | 117 | GLF | - | SCL/MADRS/HAMD | 1 | USA |
| Perini, 2009 | MDD | Adults | 8 | GSH (CBT) | 27 | 6 | WL | 18 | SDS | - | BDI-II | 3 | Australia |
| Philipp, 1999 | MDE | Adults | 8 | Imipramine | 110 | 50-100mg | Placebo | 47 | - | SF-36 | HAMD | 3 | Germany |
| Phillips, 2014 | Depression | Adults | 6 (6 week follow-up) | CCBT: MoodGym | 171 | 5 | Other | 188 | WSAS | - | PHQ-9 | 3 | UK |
| Propst, 1992 | Depression | Adults | 12 (3 and 24 monthfollow-up) | RCT-NT, RTC-RT, NRCT-RT, NRCT-NT (all individual) | 38 | 18 | WL | 11 | SAS-CR | - | HAMD | 2 | USA |
| Ransom, 2008 | MDE/Dys | Adults | 6 | IPT (telephone) | 41 | 6 | TAU | 38 | OQ-45 | - | BDI | 0 | USA |
| Rapaport, 2009 | MDD | Older Adults | 10 | Paroxetine | 138/145 | 12.5, 25mg | placebo | 150 | - | Q-LES-Q | HAMD/GDS | 3 | USA |
| Rapaport, 2011 | Minor DD | Adults | 12 | Citalopram | 24 | 20mg | placebo | 23 | GAF/MOS-36 social functioning scale | Q-LES-Q |  | 4 | USA |
| Ravindran, 1999 | Dys | Adults | 12 | CT + sertraline, CT alone (group), sertraline alone | 24/22/24 | 12/50-200mg | placebo | 24 | Batelle QLS social interaction domain | Batelle QLS | HAMD | 3 | Canada |
| Ravindran, 2000 | Dys | Adults | 12 | Sertraline | 158 | 50-200mg | placebo | 152 | - | Batelle QLS | MADRS/HAD-D | 2 | USA |
| Ravindran, 2013 | Dys | Adults | 12 | Paroxetine | 21 | 20-40mg | placebo | 19 | - | Q-LES-Q | BDI/HAMD | 2 | Canada |
| Robinson, 2000 | MDD/MinD | Adults | 12 | Nortriptyline, fluoxetine | 13/14 | 25-100 mg for nortript/ 10-40mg. for fluoxetine | placebo | 13 | Johns Hopkins FI/SFE | - | HAMD | 2 | USA |
| Rohricht, 2013 | MDD/Dys | Adults | 12 | BPT (group) | 10 | 20 | WL | 12 | - | MANSA | HAMD | 4 | UK |
| Salminen, 2008 | MDD | Adults | 16 | STPP (individual) | 26 | 16/20-40mg | fluoxetine | 25 | SOFAS | - | BDI/HAMD | 1 | Finland |
| Sambunaris, 2014 | MDD | Adults | 8 | Levomilnacipran | 181 | 20-40mg | placebo | 190 | SDS | - | MADRS/HAMD | 3 | USA |
| Sanford, 2003 | MDD | Adults | 8 | Parent-Education Group | 15 | 8 | WL | 17 | FAD | - | CES-D | 4 | Canada |
| Schneider, 2003 | MDD | Older Adults | 8 | Sertraline | 360 | 50-100mg | placebo | 368 | SF-36 social functioningscale | Q-LES-Q | HAMD | 2 | USA |
| Schramm, 2007 | MDD | Adults | 5 (3 and 12 monthfollow-up) | IPT (individual and group)/Medication | 50 | 15 individual., 8 group/ser: 90/ami:175mg | Sertraline, or amitriptyline or amitriptyline - N - Oxide | 47 | GAF | - | HAMD/BDI | 4 | Germany |
| Scott, 2000 | RD | Adults | 20 (12 monthfollow-up) | CBT+CM | 80 | 16 | CM | 78 | SAS-CR | - | BDI/HAMD/RDS | 4 | UK |
| Seligman, 2006 | MDD | Adults | 12 | PosP. (individual) | 11/12 | 14 | TAU/TAUMED | 9 | GAF | - | ZSRS/HAMD | 1 | Australia |
| Serfaty, 2009 | Depression | Older Adults | 16 (6 monthfollow-up) | CBT (individual) | 51/61 | 12 | TAU | 46/55 | SFQ | EQ-5D | BDI-II | 4 | UK |
| Serrano, 2004 | MDD | Adults | 4 (6 monthfollow-up) | LRT (individual) | 9 | 4 | Placebo | 8 | - | QLSD | GDS-15 | 2 | Spain |
| Sharp, 2010 | MDD | Women | 18 | NDC (individual) | 102 | 18 | AT | 92 | - | EQ-5D VAS | EPDS | 3 | UK |
| Simpson, 2003 | Depression | Adults | 24 (6 monthfollow-up) | PDC (individual) | 83 | 5 | TAU | 80 | IIP/SAS | - | BDI | 4 | UK |
| Stewart, 1987 | MDD/Dys | Adults | 6 | Imipramine, Phenelzine | 47/36 | 300mg. imipramine/ 90mg phenelzine | placebo | 48 | SAS-SR | - |  | 1 | USA |
| Swartz, 2008 | PPD | Women | 12 (6 monthfollow-up) | Brief IPT (individual) | 26 | 9 | TAU | 21 | GAF | - | BDI/HAMD | 1 | USA |
| Talbot, 2011 | MDD | Women | 36 | IPT (individual) | 29 | 16 | TAU | 24 | SAS-SR | MOS-36 | HAMD/BDI | 1 | USA |
| Titov, 2010 | MDE | Adults | 8 (4 monthfollow-up) | iCBT + technician, iCBT + clinician | 41/46 | 6 | WL | 40 | SDS | - | BDI-II/PHQ-9 | 4 | Australia |
| Trivedi, 2004 | MDD | Adults | 8 | paroxetine | 153/148 | 12.5, 25mg | placebo | 146 | SDS | Q-LES-Q | HAMD | 4 | USA |
| Van Aalderen, 2011 | RD | Adults | 12 | MBCT+TAU | 102 | 8 | TAU | 103 | - | WHOQOL-BREF | BDI/HAMD | 4 | Netherlands |
| Van Schaik, 2006 | MDD | Older Adults | 10 | IPT (individual) | 69 | 10 | TAU | 74 | SF-36 social functioning scale | SF-36 | MADRS/GDS | 3 | Netherlands |
| Vernmark, 2010 | MDD | Adults | 8 (6 monthfollow-up) | iGSH CBT vs. Individualized CBT Email Therapy | 29/30 | 7 | WL | 29 | - | QOLI | BDI/MADRS | 4 | Sweden |
| Vitriol, 2009 | Depression | Women | 12 (3 monthfollow-up) | PP (individual) | 44 | 12 | TAU | 43 | OQ-45 interpersonal relationships and social role | - | HAMD | 2 | Chile |
| Ward, 2000 | Depression | Adults | 16 (8 monthfollow-up) | NDC/CBT (individual) | 67/63 | 6 in both | TAU | 67 | SAS-M | - | BDI | 3 | UK |
| Watt, 2000 | Depression | Older Adults | 6 (3 monthfollow-up) | Integrative reminiscence /Instrumental reminiscence (group) | 9 | 6 | Other | 9 | SAS-SR | - | GDS/HAMD | 2 | Canada |
| Wiles, 2013 | Depression | Adults | 24 (6 monthfollow-up) | CBT+TAU (individual) | 201 | 12 | TAU | 209 | - | SF-12 | BDI | 4 | UK |
| Williams, 2013 | MDE | Adults | 11 | iCBT | 35 | 13 | WL | 28 | WHODAS | - | BDI/PHQ-9 | 2 | Australia |
| Wong, 2008 | MDD | Adults | 10 | CBT (group) | 163 | 10 | WL | 159 | - | Q-LES-Q-18 | BDI | 1 | China |
| Zilcha-Mano, 2014 | MDD | Adults | 16 | SET/sertraline | 51/55 | 20 | Placebo | 50 | IIP-64 | Q-LES-Q | BDI | 1 | USA |
| Zu, 2014 | MDD | Adults | 24 | CBT/citalopram, escitalopram, paroxetine or sertraline | 12 /25 | 20/cit:20-60; esc:10-20; par: 20-60; ser:25-100mg | TAU | 16 | WSAS | - | HAMD/QIDS-SR | 3 | China |
|  |  |  |  |  |  |  |  |  |  |  |  |  |  |

*Note: * Dosages separated by comma signify different intervention arms; ** dosages separated by dash signify varying dosage across treatment*

*ACT-E= Acceptance and commitment therapy with extensive email support, ACT-M= acceptance and commitment therapy with minimal email support, AQ=Activities Questionnaire, AT= Antidepressant Treatment, BA= behavioral activation, BAI= behavioral Activities Intervention, BDI= Beck Depression Inventory, BDI-II= BDI Second Edition, BDQ= Brief Disability Questionnaire, BDT= Brief Dynamic Therapy, BELP-KF= Berlin Quality of Life Profile, BPT= Body Psychotherapy, BSP= Brief Supportive Psychotherapy, CAU= care as usual, CBC= Cognitive Behavioral Counseling, CBT= Cognitive Behavioral therapy, CM=Clinical Management, CC=Collaborative Care, GSH= Guided Self-Help, CES-D= Center for Epidemiologic Studies Depression Scale, CIGP-CD= Cognitive-Interpersonal Group Psychotherapy for Chronic Depression, ConT= Concordance Therapy, CR= Cognitive Remediation, Cres=Cognitive Restructuring, CWD= Coping with Depression, D NOS= Depression Non otherwise Specified, Dys= Dysthymia, ECLA-T=Effectiveness of Engagement and Counseling for Latinos by Telephone; ECLA-F= Effectiveness of Engagement and Counseling for Latinos face-to-face, EPDS= Edinburgh Postnatal Depression Scale, FAD= Family Assessment Device, FDI=Functioning in Daily Activities, GDS= Geriatric Depression Scale, GLF= General Life Functioning, HAMD= Hamilton Rating Scale for Depression, HPQ= World Health Organization Health and Work Performance Questionnaire, HSQ= Health Status Questionnaire, iCBT=internet-based CBT, IIP= Inventory of Interpersonal Problems, IPA= Impact on participation and autonomy, , IPC= Interpersonal Counseling, IPRI=Interpersonal Relationships Inventory, IPT= interpersonal psychotherapy, KDQOL-SF= Kidney Disease and Quality of Life Short Form, LIFE-RIFT=The Longitudinal Interval Follow-up Evaluation Range of Impaired Functioning Tool, LRT= Life Review Therapy, MADRS= Montgomery–Åsberg Depression Rating Scale, MANSA= Manchester Short Assessment of QoL, MDD= Major Depressive Disorder, MHC-SF-SOC= Mental Health Continuum Short Form social functioning subscale, Min D=Minor Depression, MOS-SF-20= Medical Outcomes Study 20-item Short-Form Health Survey, , MPI=Minimal Psychological Intervention, NDC=Non-Directive Counseling, NRCT-RT=Nonreligious Cognitive Therapy with religious therapist, NRCT-NT= Nonreligious Cognitive Therapy with nonreligious therapist, OQ-45= Outcome Questionnaire, PDC= Psychodynamic counselling,, PI= Psychosocial Intervention, PosP=Positive Psychotherapy, PP=Psychodynamic Psychotherapy, PPD= postpartum depression, PST= problem solving therapy, QIDS= Quick Inventory of Depressive Symptomatology, QLDS= Quality of Life in Depression Scale, RCT-NT=Religious Cognitive Therapy with nonreligious therapist, RCT-RT=Religious Cognitive Therapy with religious therapist, RD= Recurrent Depression, RET=Rationale-Emotive Therapy, SAS= Social Adjustment Scale, SAS-SR= Social Adjustment Scale, Self-Report, SASS=Social Adaptation Self-Evaluation Scale, SCL-90= Symptom Checklist-90, SFE= Social Functioning Exam, SDS= Sheehan Disability Scale, SET= Supportive Expressive Therapy, SF-36= 36-item Short-Form Health Survey, SFQ= Social Functioning Questionnaire, SI= Social Intervention, SIG= Scale for Interpersonal Behavior, SOFAS= Social and Occupational Functioning Assessment Scale, SPSP= Short Psychodynamic Supportive Psychotherapy, STPP= Short Term Psychodynamic Psychotherapy, WHOQOL= World Health Organization Quality of Life, WL= waiting list, WSAS= Work and Social Adjustment Scale*

**Reference List of Included Studies**

**Agosti, V., Stewart, J. W. & Quitkin, F. M.** (1991). Life satisfaction and psychosocial functioning in chronic depression: effect of acute treatment with antidepressants. *Journal of Affective Disorders* **23**, 35-41.

**Alegria, M., Ludman, E., Kafali, E. N., Lapatin, S., Vila, D., Shrout, P. E., Keefe, K., Cook, B., Ault, A., Li, X., Bauer, A. M., Epelbaum, C., Alcantara, C., Pineda, T. I., Tejera, G. G., Suau, G., Leon, K., Lessios, A. S., Ramirez, R. R. & Canino, G.** (2014). Effectiveness of the Engagement and Counseling for Latinos (ECLA) intervention in low-income Latinos. *Medical Care* **52**, 989-97.

**Allart-van Dam, E., Hosman, C. M., Hoogduin, C. A., & & Schaap, C. P.** (2003). The coping with depression course: Short-term outcomes and mediating effects of a randomized controlled trial in the treatment of subclinical depression. *Behavioral Therapy, 34(3), 381-396.*

**Andersson, G., Bergstrom, J., Hollandare, F., Carlbring, P., Kaldo, V. & Ekselius, L.** (2005). Internet-based self-help for depression: randomised controlled trial. *British Journal of Psychiatry* **187**, 456-61.

**Araya, R., Rojas, G., Fritsch, R., Gaete, J., Rojas, M., Simon, G. & Peters, T. J.** (2003). Treating depression in primary care in low-income women in Santiago, Chile: a randomised controlled trial. *Lancet* **361**, 995-1000.

**Asnis, G. M., Bose, A., Gommoll, C. P., Chen, C. & Greenberg, W. M.** (2013). Efficacy and safety of levomilnacipran sustained release 40 mg, 80 mg, or 120 mg in major depressive disorder: a phase 3, randomized, double-blind, placebo-controlled study. *Journal of Clinical Psychiatry* **74**, 242-8.

**Bakish, D., Bose, A., Gommoll, C., Chen, C., Nunez, R., Greenberg, W. M., Liebowitz, M. & Khan, A.** (2014). Levomilnacipran ER 40 mg and 80 mg in patients with major depressive disorder: a phase III, randomized, double-blind, fixed-dose, placebo-controlled study. *Journal of Psychiatry and Neuroscience* **39**, 40-9.

**Barge-Schaapveld, D. Q. & Nicolson, N. A.** (2002). Effects of antidepressant treatment on the quality of daily life: an experience sampling study. *Journal of Clinical Psychiatry* **63**, 477-85.

**Barrett, J. E., Williams, J. W., Jr., Oxman, T. E., Frank, E., Katon, W., Sullivan, M., Hegel, M. T., Cornell, J. E. & Sengupta, A. S.** (2001). Treatment of dysthymia and minor depression in primary care: a randomized trial in patients aged 18 to 59 years. *Journal of Family Practice* **50**, 405-12.

**Bass, J., Neugebauer, R., Clougherty, K. F., Verdeli, H., Wickramaratne, P., Ndogoni, L., Speelman, L., Weissman, M. & Bolton, P.** (2006). Group interpersonal psychotherapy for depression in rural Uganda: 6-month outcomes: randomised controlled trial. *Bitishr Journal of Psychiatry* **188**, 567-73.

**Bedi, N., Chilvers, C., Churchill, R., Dewey, M., Duggan, C., Fielding, K., Gretton, V., Miller, P., Harrison, G., Lee, A. & Williams, I.** (2000). Assessing effectiveness of treatment of depression in primary care. Partially randomised preference trial. *British Journal of Psychiatry* **177**, 312-8.

**Bellack, A. S., Hersen, M. & Himmelhoch, J.** (1981). Social skills training compared with pharmacotherapy and psychotherapy in the treatment of unipolar depression. *American Journal of Psychiatry* **138**, 1562-7.

**Bellino, S., Zizza, M., Rinaldi, C. & Bogetto, F.** (2006). Combined treatment of major depression in patients with borderline personality disorder: a comparison with pharmacotherapy. *Canadian Journal of Psychiatry* **51**, 453-60.

**Berger, T., Hammerli, K., Gubser, N., Andersson, G. & Caspar, F.** (2011). Internet-based treatment of depression: a randomized controlled trial comparing guided with unguided self-help. *Cognitive Behavioral Therapy* **40**, 251-66.

**Blum, S. I., Tourkodimitris, S. & Ruth, A.** (2015). Evaluation of functional health and well-being in patients receiving levomilnacipran ER for the treatment of major depressive disorder. *Journal of Affect Disord* **170**, 230-6.

**Bowie, C. R., Gupta, M., Holshausen, K., Jokic, R., Best, M. & Milev, R.** (2013). Cognitive remediation for treatment-resistant depression: effects on cognition and functioning and the role of online homework. *Journal of Nervous and Mental Disease* **201**, 680-5.

**Boyer, P., Montgomery, S., Lepola, U., Germain, J. M., Brisard, C., Ganguly, R., Padmanabhan, S. K. & Tourian, K. A.** (2008). Efficacy, safety, and tolerability of fixed-dose desvenlafaxine 50 and 100 mg/day for major depressive disorder in a placebo-controlled trial. *International Clinical Psychopharmacology* **23**, 243-53.

**Brown, C., Schulberg, H. C., Madonia, M. J., Shear, M. K. & Houck, P. R.** (1996). Treatment outcomes for primary care patients with major depression and lifetime anxiety disorders. *American Journal of Psychiatry* **153**, 1293-300.

**Browne, G., Steiner, M., Roberts, J., Gafni, A., Byrne, C., Dunn, E., Bell, B., Mills, M., Chalklin, L., Wallik, D. & Kraemer, J.** (2002). Sertraline and/or interpersonal psychotherapy for patients with dysthymic disorder in primary care: 6-month comparison with longitudinal 2-year follow-up of effectiveness and costs. *Journal of Affective Disorders* **68**, 317-30.

**Burke, W. J., Gergel, I. & Bose, A.** (2002). Fixed-dose trial of the single isomer SSRI escitalopram in depressed outpatients. *Journal of Clinical Psychiatry* **63**, 331-6.

**Burnand, Y., Andreoli, A., Kolatte, E., Venturini, A. & Rosset, N.** (2002). Psychodynamic psychotherapy and clomipramine in the treatment of major depression. *Psychiatr ic Service* **53**, 585-90.

**Burt, V. K., Wohlreich, M. M., Mallinckrodt, C. H., Detke, M. J., Watkin, J. G. & Stewart, D. E.** (2005). Duloxetine for the treatment of major depressive disorder in women ages 40 to 55 years. *Psychosomatics* **46**, 345-54.

**Carlbring, P., Hagglund, M., Luthstrom, A., Dahlin, M., Kadowaki, A., Vernmark, K. & Andersson, G.** (2013). Internet-based behavioral activation and acceptance-based treatment for depression: a randomized controlled trial. *Journal of Affective Disorders* **148**, 331-7.

**Carpenter, L. L., Yasmin, S. & Price, L. H.** (2002). A double-blind, placebo-controlled study of antidepressant augmentation with mirtazapine. *Bioloogical Psychiatry* **51**, 183-8.

**Carta, M., Petretto, D., Adamo, S., Bhat, K., Lecca, M., Mura, G., Carta, V., Angermeyer, M. & Moro, M.** (2012). Counseling in primary care improves depression and quality of life. *Clinical Practice and Epidemiology of Mental Health* **8**, 152-7.

**Casanas, R., Catalan, R., del Val, J. L., Real, J., Valero, S. & Casas, M.** (2012). Effectiveness of a psycho-educational group program for major depression in primary care: a randomized controlled trial. *BMC Psychiatry* **12**, 230.

**Chaput, Y., Magnan, A. & Gendron, A.** (2008). The co-administration of quetiapine or placebo to cognitive-behavior therapy in treatment refractory depression: a preliminary trial. *BMC Psychiatry* **8**, 73.

**Choi, I., Zou, J., Titov, N., Dear, B. F., Li, S., Johnston, L., Andrews, G. & Hunt, C.** (2012). Culturally attuned Internet treatment for depression amongst Chinese Australians: a randomised controlled trial. *Journal of Affective Disorders* **136**, 459-68.

**Clarke, G., Eubanks, D., Reid, E., Kelleher, C., O'Connor, E., DeBar, L. L., Lynch, F., Nunley, S. & Gullion, C.** (2005). Overcoming Depression on the Internet (ODIN) (2): a randomized trial of a self-help depression skills program with reminders. *Journal of Medical and Internet Research* **7**, e16.

**Clayton, A. H., Kornstein, S. G., Dunlop, B. W., Focht, K., Musgnung, J., Ramey, T., Bao, W. & Ninan, P. T.** (2013). Efficacy and safety of desvenlafaxine 50 mg/d in a randomized, placebo-controlled study of perimenopausal and postmenopausal women with major depressive disorder. *Journal of Clinical Psychiatry* **74**, 1010-7.

**Coventry, P., Lovell, K., Dickens, C., Bower, P., Chew-Graham, C., McElvenny, D., Hann, M., Cherrington, A., Garrett, C., Gibbons, C. J., Baguley, C., Roughley, K., Adeyemi, I., Reeves, D., Waheed, W. & Gask, L.** (2015). Integrated primary care for patients with mental and physical multimorbidity: cluster randomised controlled trial of collaborative care for patients with depression comorbid with diabetes or cardiovascular disease. *BMJ* **350**, h638.

**Cramer, H., Salisbury, C., Conrad, J., Eldred, J. & Araya, R.** (2011). Group cognitive behavioural therapy for women with depression: pilot and feasibility study for a randomised controlled trial using mixed methods. *BMC Psychiatry* **11**, 82.

**de Graaf, L. E., Gerhards, S. A., Arntz, A., Riper, H., Metsemakers, J. F., Evers, S. M., Severens, J. L., Widdershoven, G. & Huibers, M. J.** (2009). Clinical effectiveness of online computerised cognitive-behavioural therapy without support for depression in primary care: randomised trial. *British Journal of Psychiatry* **195**, 73-80.

**de Jonghe, F., Kool, S., van Aalst, G., Dekker, J. & Peen, J.** (2001). Combining psychotherapy and antidepressants in the treatment of depression. *Journal of Affective Disorders* **64**, 217-29.

**de Mello, M. F., Myczcowisk, L. M. & Menezes, P. R.** (2001). A randomized controlled trial comparing moclobemide and moclobemide plus interpersonal psychotherapy in the treatment of dysthymic disorder. *Journal of Psychotheraphy Practice Research* **10**, 117-23.

**Detke, M. J., Lu, Y., Goldstein, D. J., Hayes, J. R. & Demitrack, M. A.** (2002a). Duloxetine, 60 mg once daily, for major depressive disorder: a randomized double-blind placebo-controlled trial. *Journal of Clinical Psychiatry* **63**, 308-15.

**Detke, M. J., Lu, Y., Goldstein, D. J., McNamara, R. K. & Demitrack, M. A.** (2002b). Duloxetine 60 mg once daily dosing versus placebo in the acute treatment of major depression. *Journal of Psychiatric Research* **36**, 383-90.

**Devanand, D. P., Nobler, M. S., Cheng, J., Turret, N., Pelton, G. H., Roose, S. P. & Sackeim, H. A.** (2005). Randomized, double-blind, placebo-controlled trial of fluoxetine treatment for elderly patients with dysthymic disorder. *American Journal of Geriatric Psychiatry* **13**, 59-68.

**Dobkin, R. D., Menza, M., Allen, L. A., Gara, M. A., Mark, M. H., Tiu, J., Bienfait, K. L. & Friedman, J.** (2011). Cognitive-behavioral therapy for depression in Parkinson's disease: a randomized, controlled trial. *American Journal of Psychiatry* **168**, 1066-74.

**Dowrick, C., Dunn, G., Ayuso-Mateos, J. L., Dalgard, O. S., Page, H., Lehtinen, V., Casey, P., Wilkinson, C., Vazquez-Barquero, J. L. & Wilkinson, G.** (2000). Problem solving treatment and group psychoeducation for depression: multicentre randomised controlled trial. Outcomes of Depression International Network (ODIN) Group. *BMJ* **321**, 1450-4.

**Duarte, P. S., Miyazaki, M. C., Blay, S. L. & Sesso, R.** (2009). Cognitive-behavioral group therapy is an effective treatment for major depression in hemodialysis patients. *Kidney International* **76**, 414-21.

**Dubini, A., Bosc, M. & Polin, V.** (1997). Noradrenaline-selective versus serotonin-selective antidepressant therapy: differential effects on social functioning. *Journal of Psychopharmacol* ogy **11**, S17-23.

**Dunlop, B. W., Reddy, S., Yang, L., Lubaczewski, S., Focht, K. & Guico-Pabia, C. J.** (2011). Symptomatic and functional improvement in employed depressed patients: a double-blind clinical trial of desvenlafaxine versus placebo. *Journal of Clinical Psychopharmacology* **31**, 569-76.

**Ekers, D., Richards, D., McMillan, D., Bland, J. M. & Gilbody, S.** (2011). Behavioural activation delivered by the non-specialist: phase II randomised controlled trial. *British Journal of Psychiatry* **198**, 66-72.

**Elkin, I., Shea, M. T., Watkins, J. T., Imber, S. D., Sotsky, S. M., Collins, J. F., Glass, D. R., Pilkonis, P. A., Leber, W. R., Docherty, J. P. & et al.** (1989). National Institute of Mental Health Treatment of Depression Collaborative Research Program. General effectiveness of treatments. *Archives of General Psychiatry* **46**, 971-82; discussion 983.

**Faramarzi, M., Kheirkhah, F., Esmaelzadeh, S., Alipour, A., Hjiahmadi, M. & Rahnama, J.** (2008). Is psychotherapy a reliable alternative to pharmacotherapy to promote the mental health of infertile women? A randomized clinical trial. *European Journal of Obstetric Gynecology and Reproductive Biol*ogy **141**, 49-53.

**Fledderus, M., Bohlmeijer, E. T., Pieterse, M. E. & Schreurs, K. M.** (2012). Acceptance and commitment therapy as guided self-help for psychological distress and positive mental health: a randomized controlled trial. *Psychological Medicine* **42**, 485-95.

**Furukawa, T. A., Horikoshi, M., Kawakami, N., Kadota, M., Sasaki, M., Sekiya, Y., Hosogoshi, H., Kashimura, M., Asano, K., Terashima, H., Iwasa, K., Nagasaku, M. & Grothaus, L. C.** (2012). Telephone cognitive-behavioral therapy for subthreshold depression and presenteeism in workplace: a randomized controlled trial. *PLoS One* **7**, e35330.

**Gater, R., Waheed, W., Husain, N., Tomenson, B., Aseem, S. & Creed, F.** (2010). Social intervention for British Pakistani women with depression: randomised controlled trial. *British Journal of Psychiatry* **197**, 227-33.

**Gaynor, P. J., Gopal, M., Zheng, W., Martinez, J. M., Robinson, M. J. & Marangell, L. B.** (2011). A randomized placebo-controlled trial of duloxetine in patients with major depressive disorder and associated painful physical symptoms. *Curr ent Medical Research Opinion* **27**, 1849-58.

**Gellis, Z. D., McGinty, J., Tierney, L., Jordan, C., Burton, J., & & Misener, E.** (2008). Randomized controlled trial of problem-solving therapy for minor depression in home care. *Research on Social Work Practice, 18(6), 596-606.*

**Goldstein, D. J., Lu, Y., Detke, M. J., Wiltse, C., Mallinckrodt, C. & Demitrack, M. A.** (2004). Duloxetine in the treatment of depression: a double-blind placebo-controlled comparison with paroxetine. *Journal of Clinical Psychopharmacology* **24**, 389-99.

**Gottlieb, S. S., Kop, W. J., Thomas, S. A., Katzen, S., Vesely, M. R., Greenberg, N., Marshall, J., Cines, M. & Minshall, S.** (2007). A double-blind placebo-controlled pilot study of controlled-release paroxetine on depression and quality of life in chronic heart failure. *American Heart Journal* **153**, 868-73.

**Grote, N. K., Swartz, H. A., Geibel, S. L., Zuckoff, A., Houck, P. R. & Frank, E.** (2009). A randomized controlled trial of culturally relevant, brief interpersonal psychotherapy for perinatal depression. *Psychiatric Services* **60**, 313-21.

**Group., H. D. T. S.** (2002). Effect of Hypericum perforatum (St John's wort) in major depressive disorder: a randomized controlled trial. *JAMA* **287**, 1807-14.

**Haringsma, R., Engels, G. I., Cuijpers, P. & Spinhoven, P.** (2006). Effectiveness of the Coping With Depression (CWD) course for older adults provided by the community-based mental health care system in the Netherlands: a randomized controlled field trial. *International Psychogeriatry* **18**, 307-25.

**Harley, R., Sprich, S., Safren, S., Jacobo, M. & Fava, M.** (2008). Adaptation of dialectical behavior therapy skills training group for treatment-resistant depression. *J ournal of Nervous and Mental Disease* **196**, 136-43.

**Healy, D.** (2000). Reboxetine: its effects as measured by the Social Adaptation Self-evaluation Scale. *Acta Psychiatrica Scandinavica Suppl* **402**, 45-51.

**Heiligenstein, J. H., Ware, J. E., Jr., Beusterien, K. M., Roback, P. J., Andrejasich, C. & Tollefson, G. D.** (1995). Acute effects of fluoxetine versus placebo on functional health and well-being in late-life depression. *International Psychogeriatry* **7 Suppl**, 125-37.

**Hellerstein, D. J., Batchelder, S. T., Hyler, S., Arnaout, B., Toba, C., Benga, I. & Gangure, D.** (2010). Escitalopram versus placebo in the treatment of dysthymic disorder. *International Clinical Psychopharmacol* ogy**25**, 143-8.

**Hellerstein, D. J., Little, S. A., Samstag, L. W., Batchelder, S., Muran, J. C., Fedak, M., Kreditor, D., Rosenthal, R. N. & Winston, A.** (2001). Adding group psychotherapy to medication treatment in dysthymia: a randomized prospective pilot study. *Journal of Psychotherapy and Practice Research* **10**, 93-103.

**Hellerstein, D. J., Stewart, J. W., McGrath, P. J., Deliyannides, D. A., Batchelder, S. T., Black, S. R., Withers, A., O'Shea, D. & Chen, Y.** (2012). A randomized controlled trial of duloxetine versus placebo in the treatment of nonmajor chronic depression. *Journal of Clinical Psychiatry* **73**, 984-91.

**Hewett, K., Chrzanowski, W., Jokinen, R., Felgentreff, R., Shrivastava, R. K., Gee, M. D., Wightman, D. S., O'Leary, M. C., Millen, L. S., Leon, M. C., Briggs, M. A., Krishen, A. & Modell, J. G.** (2010). Double-blind, placebo-controlled evaluation of extended-release bupropion in elderly patients with major depressive disorder. *J ournal of Psychopharmacology* **24**, 521-9.

**Hewett, K., Chrzanowski, W., Schmitz, M., Savela, A., Milanova, V., Gee, M., Krishen, A., Millen, L., Leary, M. O. & Modell, J.** (2009). Eight-week, placebo-controlled, double-blind comparison of the antidepressant efficacy and tolerability of bupropion XR and venlafaxine XR. *J Psychopharmacol* ogy**23**, 531-8.

**Higgins, N., Livingston, G. & Katona, C.** (2004). Concordance therapy: an intervention to help older people take antidepressants. *Journal of Affective Disorders* **81**, 287-91.

**Hirschfeld, R. M., Dunner, D. L., Keitner, G., Klein, D. N., Koran, L. M., Kornstein, S. G., Markowitz, J. C., Miller, I., Nemeroff, C. B., Ninan, P. T., Rush, A. J., Schatzberg, A. F., Thase, M. E., Trivedi, M. H., Borian, F. E., Crits-Christoph, P. & Keller, M. B.** (2002). Does psychosocial functioning improve independent of depressive symptoms? A comparison of nefazodone, psychotherapy, and their combination. *Biol ogical Psychiatry* **51**, 123-33.

**Hoifodt, R. S., Lillevoll, K. R., Griffiths, K. M., Wilsgaard, T., Eisemann, M., Waterloo, K. & Kolstrup, N.** (2013). The clinical effectiveness of web-based cognitive behavioral therapy with face-to-face therapist support for depressed primary care patients: randomized controlled trial. *Journal of Medical Internet Research* **15**, e153.

**Hollon, S. D., DeRubeis, R. J., Evans, M. D., Wiemer, M. J., Garvey, M. J., Grove, W. M. & Tuason, V. B.** (1992). Cognitive therapy and pharmacotherapy for depression. Singly and in combination. *Archives of General Psychiatry* **49**, 774-81.

**Husain, N., Chaudhry, N., Fatima, B., Husain, M., Amin, R., Chaudhry, I. B., Ur Rahman, R., Tomenson, B., Jafri, F., Naeem, F. & Creed, F.** (2014). Antidepressant and group psychosocial treatment for depression: a rater blind exploratory RCT from a low income country. *Behavioral Cognitive Psychotherapy* **42**, 693-705.

**Iwata, N., Tourian, K. A., Hwang, E., Mele, L. & Vialet, C.** (2013). Efficacy and safety of desvenlafaxine 25 and 5050% shaded blockmg/day in a randomized, placebo-controlled study of depressed outpatients. *J ournal of Psychiatric Practice* **19**, 5-14.

**Johansson, R., Sjoberg, E., Sjogren, M., Johnsson, E., Carlbring, P., Andersson, T., Rousseau, A. & Andersson, G.** (2012). Tailored vs. standardized internet-based cognitive behavior therapy for depression and comorbid symptoms: a randomized controlled trial. *PLoS One* **7**, e36905.

**Jonkers, C. C., Lamers, F., Bosma, H., Metsemakers, J. F. & van Eijk, J. T.** (2012). The effectiveness of a minimal psychological intervention on self-management beliefs and behaviors in depressed chronically ill elderly persons: a randomized trial. *International Psychogeriatrics* **24**, 288-97.

**Judd, F. K., Piterman, L., Cockram, A. M., McCall, L. & Weissman, M. M.** (2001). A comparative study of venlafaxine with a focused education and psychotherapy program versus venlafaxine alone in the treatment of depression in general practice. *Human Psychopharmacol* ogy **16**, 423-428.

**Judd, L. L., Rapaport, M. H., Yonkers, K. A., Rush, A. J., Frank, E., Thase, M. E., Kupfer, D. J., Plewes, J. M., Schettler, P. J. & Tollefson, G.** (2004). Randomized, placebo-controlled trial of fluoxetine for acute treatment of minor depressive disorder. *American Journal of Psychiatry* **161**, 1864-71.

**Kanter, J. W., Santiago-Rivera, A. L., Santos, M. M., Nagy, G., Lopez, M., Hurtado, G. D. & West, P.** (2015). A randomized hybrid efficacy and effectiveness trial of behavioral activation for Latinos with depression. *Behavioral Therapy* **46**, 177-92.

**Keitner, G. I., Garlow, S. J., Ryan, C. E., Ninan, P. T., Solomon, D. A., Nemeroff, C. B. & Keller, M. B.** (2009). A randomized, placebo-controlled trial of risperidone augmentation for patients with difficult-to-treat unipolar, non-psychotic major depression. *Journal of Psychiatric Research* **43**, 205-14.

**Kessler, D., Lewis, G., Kaur, S., Wiles, N., King, M., Weich, S., Sharp, D. J., Araya, R., Hollinghurst, S. & Peters, T. J.** (2009). Therapist-delivered Internet psychotherapy for depression in primary care: a randomised controlled trial. *Lancet* **374**, 628-34.

**Kim, J. M., Bae, K. Y., Stewart, R., Jung, B. O., Kang, H. J., Kim, S. W., Shin, I. S., Hong, Y. J., Kim, J. H., Shin, H. Y., Kang, G., Ahn, Y., Kim, J. K., Jeong, M. H. & Yoon, J. S.** (2015). Escitalopram treatment for depressive disorder following acute coronary syndrome: a 24-week double-blind, placebo-controlled trial. *Journal of Clinical Psychiatry* **76**, 62-8.

**Klug, G., Hermann, G., Fuchs-Nieder, B., Panzer, M., Haider-Stipacek, A., Zapotoczky, H. G. & Priebe, S.** (2010). Effectiveness of home treatment for elderly people with depression: randomised controlled trial. *British Journal of Psychiatry* **197**, 463-7.

**Kocsis, J. H., Frances, A. J., Voss, C., Mann, J. J., Mason, B. J. & Sweeney, J.** (1988). Imipramine treatment for chronic depression. *Arch ives of General Psychiatry* **45**, 253-7.

**Kocsis, J. H., Zisook, S., Davidson, J., Shelton, R., Yonkers, K., Hellerstein, D. J., Rosenbaum, J. & Halbreich, U.** (1997). Double-blind comparison of sertraline, imipramine, and placebo in the treatment of dysthymia: psychosocial outcomes. *American Journal of Psychiatry* **154**, 390-5.

**Korte, J., Bohlmeijer, E. T., Cappeliez, P., Smit, F. & Westerhof, G. J.** (2012). Life review therapy for older adults with moderate depressive symptomatology: a pragmatic randomized controlled trial. *Psychol ogical Medicine*  **42**, 1163-73.

**Laidlaw, K., Davidson, K., Toner, H., Jackson, G., Clark, S., Law, J., Howley, M., Bowie, G., Connery, H. & Cross, S.** (2008). A randomised controlled trial of cognitive behaviour therapy vs treatment as usual in the treatment of mild to moderate late life depression. *International Journal of Geriatric Psychiatry* **23**, 843-50.

**Lenderking, W. R., Tennen, H., Nackley, J. F., Hale, M. S., Turner, R. R. & Testa, M. A.** (1999). The effects of venlafaxine on social activity level in depressed outpatients. *Journal of Clinical Psychiatry* **60**, 157-63.

**Lesperance, F., Frasure-Smith, N., Koszycki, D., Laliberte, M. A., van Zyl, L. T., Baker, B., Swenson, J. R., Ghatavi, K., Abramson, B. L., Dorian, P. & Guertin, M. C.** (2007). Effects of citalopram and interpersonal psychotherapy on depression in patients with coronary artery disease: the Canadian Cardiac Randomized Evaluation of Antidepressant and Psychotherapy Efficacy (CREATE) trial. *JAMA* **297**, 367-79.

**Liebowitz, M. R., Manley, A. L., Padmanabhan, S. K., Ganguly, R., Tummala, R. & Tourian, K. A.** (2008). Efficacy, safety, and tolerability of desvenlafaxine 50 mg/day and 100 mg/day in outpatients with major depressive disorder. *Current Medical Research Opinion* **24**, 1877-90.

**Liebowitz, M. R., Tourian, K. A., Hwang, E. & Mele, L.** (2013). A double-blind, randomized, placebo-controlled study assessing the efficacy and tolerability of desvenlafaxine 10 and 50 mg/day in adult outpatients with major depressive disorder. *BMC Psychiatry* **13**, 94.

**Locklear, J. C., Svedsater, H., Datto, C. & Endicott, J.** (2013). Effects of once-daily extended release quetiapine fumarate (quetiapine XR) on quality of life and sleep in elderly patients with major depressive disorder. *Journal of Affective Disorders* **149**, 189-95.

**Lydiard, R. B., Stahl, S. M., Hertzman, M. & Harrison, W. M.** (1997). A double-blind, placebo-controlled study comparing the effects of sertraline versus amitriptyline in the treatment of major depression. *Journal of Clinical Psychiatry* **58**, 484-91.

**Lynch, D., Tamburrino, M., Nagel, R. & Smith, M. K.** (2004). Telephone-based treatment for family practice patients with mild depression. *Psychological Rep* **94**, 785-92.

**Lynch, D. J., Tamburrino, M. B. & Nagel, R.** (1997). Telephone counseling for patients with minor depression: preliminary findings in a family practice setting. *Journal of Family Practice* **44**, 293-8.

**Macaskill ND & Macaskill, A.** (1996). Rational-Emotive Therapy Plus Pharmacotherapy Versus Pharmacotherapy Alone in the Treatment of High Cognitive Dysfunction Depression *Cognitive Therapy and Research* **20**, 575-592.

**Mahmoud, R. A., Pandina, G. J., Turkoz, I., Kosik-Gonzalez, C., Canuso, C. M., Kujawa, M. J. & Gharabawi-Garibaldi, G. M.** (2007). Risperidone for treatment-refractory major depressive disorder: a randomized trial. *Annals of Internal Medicine* **147**, 593-602.

**Maina, G., Rosso, G., Rigardetto, S., Chiado Piat, S. & Bogetto, F.** (2010). No effect of adding brief dynamic therapy to pharmacotherapy in the treatment of obsessive-compulsive disorder with concurrent major depression. *Psychotherapy and Psychosomatics* **79**, 295-302.

**Markowitz, J. C., Kocsis, J. H., Bleiberg, K. L., Christos, P. J. & Sacks, M.** (2005). A comparative trial of psychotherapy and pharmacotherapy for "pure" dysthymic patients. *Journal of Affective Disorders* **89**, 167-75.

**McIntyre, A., Paisley, D., Kouassi, E. & Gendron, A.** (2014). Quetiapine fumarate extended-release for the treatment of major depression with comorbid fibromyalgia syndrome: a double-blind, randomized, placebo-controlled study. *Arthritis Rheumatol* ogy **66**, 451-61.

**Meeks, S., Van Haitsma, K., Schoenbachler, B. & Looney, S. W.** (2015). BE-ACTIV for depression in nursing homes: primary outcomes of a randomized clinical trial. *Journal of Gerontology B Psychological Science and Social Sciennce* **70**, 13-23.

**Miller, I. W., Norman, W. H. & Keitner, G. I.** (1999). Combined treatment for patients with double depression. *Psychotherapy and Psychosomatics* **68**, 180-5.

**Miller, L. & Weissman, M.** (2002). Interpersonal psychotherapy delivered over the telephone to recurrent depressives. A pilot study. *Depression and Anxiety* **16**, 114-7.

**Miranda, J., Chung, J. Y., Green, B. L., Krupnick, J., Siddique, J., Revicki, D. A. & Belin, T.** (2003). Treating depression in predominantly low-income young minority women: a randomized controlled trial. *JAMA* **290**, 57-65.

**Montgomery, S. A., Mansuy, L., Ruth, A., Bose, A., Li, H. & Li, D.** (2013). Efficacy and safety of levomilnacipran sustained release in moderate to severe major depressive disorder: a randomized, double-blind, placebo-controlled, proof-of-concept study. *J ournal of Clinical Psychiatry* **74**, 363-9.

**Moritz, S., Schilling, L., Hauschildt, M., Schroder, J. & Treszl, A.** (2012). A randomized controlled trial of internet-based therapy in depression. *Behavioral Research Therapy* **50**, 513-21.

**Mynors-Wallis, L. M., Gath, D. H., Day, A. & Baker, F.** (2000). Randomised controlled trial of problem solving treatment, antidepressant medication, and combined treatment for major depression in primary care. *BMJ* **320**, 26-30.

**Mynors-Wallis, L. M., Gath, D. H., Lloyd-Thomas, A. R. & Tomlinson, D.** (1995). Randomised controlled trial comparing problem solving treatment with amitriptyline and placebo for major depression in primary care. *BMJ* **310**, 441-5.

**Naeem, F., Sarhandi, I., Gul, M., Khalid, M., Aslam, M., Anbrin, A., Saeed, S., Noor, M., Fatima, G., Minhas, F., Husain, N. & Ayub, M.** (2014). A multicentre randomised controlled trial of a carer supervised culturally adapted CBT (CaCBT) based self-help for depression in Pakistan. *Journal of Affective Disorders* **56**, 224-7.

**Neugebauer, R., Kline, J., Markowitz, J. C., Bleiberg, K. L., Baxi, L., Rosing, M. A., Levin, B. & Keith, J.** (2006). Pilot randomized controlled trial of interpersonal counseling for subsyndromal depression following miscarriage. *Journal of Clinical Psychiatry* **67**, 1299-304.

**Nickel, C., Lahmann, C., Tritt, K., Muehlbacher, M., Kaplan, P., Kettler, C., Krawczyk, J., Loew, T. H., Rother, W. K. & Nickel, M. K.** (2005). Topiramate in treatment of depressive and anger symptoms in female depressive patients: a randomized, double-blind, placebo-controlled study. *Journal of Affective Disorders* **87**, 243-52.

**O'Hara, M. W., Stuart, S., Gorman, L. L. & Wenzel, A.** (2000). Efficacy of interpersonal psychotherapy for postpartum depression. *Archives of General Psychiatry* **57**, 1039-45.

**Oakes, T. M., Myers, A. L., Marangell, L. B., Ahl, J., Prakash, A., Thase, M. E. & Kornstein, S. G.** (2012). Assessment of depressive symptoms and functional outcomes in patients with major depressive disorder treated with duloxetine versus placebo: primary outcomes from two trials conducted under the same protocol. *Human Psychopharmacology* **27**, 47-56.

**Pangallo, B., Dellva, M. A., D'Souza, D. N., Essink, B., Russell, J. & Goldberger, C.** (2011). A randomized, double-blind study comparing LY2216684 and placebo in the treatment of major depressive disorder. *Journal of Psychiatric Research* **45**, 748-55.

**Pedersen, R. D., Pallay, A. G. & Rudolph, R. L.** (2002). Can improvement in well-being and functioning be distinguished from depression improvement in antidepressant clinical trials? *Quality of Life Reseaarch* **11**, 9-17.

**Perini, S., Titov, N. & Andrews, G.** (2009). Clinician-assisted Internet-based treatment is effective for depression: randomized controlled trial. *Aust ralian and New Zealand Journal of Psychiatry* **43**, 571-8.

**Philipp, M., Kohnen, R. & Hiller, K. O.** (1999). Hypericum extract versus imipramine or placebo in patients with moderate depression: randomised multicentre study of treatment for eight weeks. *BMJ* **319**, 1534-8.

**Phillips, R., Schneider, J., Molosankwe, I., Leese, M., Foroushani, P. S., Grime, P., McCrone, P., Morriss, R. & Thornicroft, G.** (2014). Randomized controlled trial of computerized cognitive behavioural therapy for depressive symptoms: effectiveness and costs of a workplace intervention. *Psychol ogical Medicine*  **44**, 741-52.

**Propst, L. R., Ostrom, R., Watkins, P., Dean, T. & Mashburn, D.** (1992). Comparative efficacy of religious and nonreligious cognitive-behavioral therapy for the treatment of clinical depression in religious individuals. *Journal of Consulting and Clinical Psychology*  **60**, 94-103.

**Ransom, D., Heckman, T. G., Anderson, T., Garske, J., Holroyd, K. & Basta, T.** (2008). Telephone-delivered, interpersonal psychotherapy for HIV-infected rural persons with depression: a pilot trial. *Psychiatr ic Services* **59**, 871-7.

**Rapaport, M. H., Lydiard, R. B., Pitts, C. D., Schaefer, D., Bartolic, E. I., Iyengar, M., Carfagno, M. & Lipschitz, A.** (2009). Low doses of controlled-release paroxetine in the treatment of late-life depression: a randomized, placebo-controlled trial. *Journal of Clinical Psychiatry* **70**, 46-57.

**Rapaport, M. H., Nierenberg, A. A., Howland, R., Dording, C., Schettler, P. J. & Mischoulon, D.** (2011). The treatment of minor depression with St. John's Wort or citalopram: failure to show benefit over placebo. *J ournal of Psychiatric Research* **45**, 931-41.

**Ravindran, A. V., Anisman, H., Merali, Z., Charbonneau, Y., Telner, J., Bialik, R. J., Wiens, A., Ellis, J. & Griffiths, J.** (1999). Treatment of primary dysthymia with group cognitive therapy and pharmacotherapy: clinical symptoms and functional impairments. *American Journal of Psychiatry* **156**, 1608-17.

**Ravindran, A. V., Cameron, C., Bhatla, R., Ravindran, L. N. & da Silva, T. L.** (2013). Paroxetine in the treatment of dysthymic disorder without co-morbidities: A double-blind, placebo-controlled, flexible-dose study. *Asian Journal of Psychiatr* **6**, 157-61.

**Ravindran, A. V., Guelfi, J. D., Lane, R. M. & Cassano, G. B.** (2000). Treatment of dysthymia with sertraline: a double-blind, placebo-controlled trial in dysthymic patients without major depression. *Journal of Clinical Psychiatry* **61**, 821-7.

**Robinson, R. G., Schultz, S. K., Castillo, C., Kopel, T., Kosier, J. T., Newman, R. M., Curdue, K., Petracca, G. & Starkstein, S. E.** (2000). Nortriptyline versus fluoxetine in the treatment of depression and in short-term recovery after stroke: a placebo-controlled, double-blind study. *American Journal of Psychiatry* **157**, 351-9.

**Rohricht, F., Papadopoulos, N. & Priebe, S.** (2013). An exploratory randomized controlled trial of body psychotherapy for patients with chronic depression. *Journal of Affective Disorders* **151**, 85-91.

**Salminen, J. K., Karlsson, H., Hietala, J., Kajander, J., Aalto, S., Markkula, J., Rasi-Hakala, H. & Toikka, T.** (2008). Short-term psychodynamic psychotherapy and fluoxetine in major depressive disorder: a randomized comparative study. *Psychotherapy and Psychosomatics* **77**, 351-7.

**Sambunaris, A., Bose, A., Gommoll, C. P., Chen, C., Greenberg, W. M. & Sheehan, D. V.** (2014). A phase III, double-blind, placebo-controlled, flexible-dose study of levomilnacipran extended-release in patients with major depressive disorder. *Journal of Clinical Psychopharmacology* **34**, 47-56.

**Sanford, M., Byrne, C., Williams, S., Atley, S., Miller, J. & Allin, H.** (2003). A pilot study of a parent-education group for families affected by depression. *Canadian Journal of Psychiatry* **48**, 78-86.

**Schneider, L. S., Nelson, J. C., Clary, C. M., Newhouse, P., Krishnan, K. R., Shiovitz, T. & Weihs, K.** (2003). An 8-week multicenter, parallel-group, double-blind, placebo-controlled study of sertraline in elderly outpatients with major depression. *American Journal of Psychiatry* **160**, 1277-85.

**Schramm, E., van Calker, D., Dykierek, P., Lieb, K., Kech, S., Zobel, I., Leonhart, R. & Berger, M.** (2007). An intensive treatment program of interpersonal psychotherapy plus pharmacotherapy for depressed inpatients: acute and long-term results. *American Journal of Psychiatry* **164**, 768-77.

**Scott, J., Teasdale, J. D., Paykel, E. S., Johnson, A. L., Abbott, R., Hayhurst, H., Moore, R. & Garland, A.** (2000). Effects of cognitive therapy on psychological symptoms and social functioning in residual depression. *British Journal of Psychiatry* **177**, 440-6.

**Seligman, M. E., Rashid, T. & Parks, A. C.** (2006). Positive psychotherapy. *American Psychol* ogy **61**, 774-88.

**Serfaty, M. A., Haworth, D., Blanchard, M., Buszewicz, M., Murad, S. & King, M.** (2009). Clinical effectiveness of individual cognitive behavioral therapy for depressed older people in primary care: a randomized controlled trial. *Archives of General Psychiatry* **66**, 1332-40.

**Serrano Selva, J. P., Latorre Postigo, J. M., Ros Segura, L., Navarro Bravo, B., Aguilar Corcoles, M. J., Nieto Lopez, M., Ricarte Trives, J. J. & Gatz, M.** (2012). Life review therapy using autobiographical retrieval practice for older adults with clinical depression. *Psicothema* **24**, 224-9.

**Sharp, D. J., Chew-Graham, C., Tylee, A., Lewis, G., Howard, L., Anderson, I., Abel, K., Turner, K. M., Hollinghurst, S. P., Tallon, D., McCarthy, A. & Peters, T. J.** (2010). A pragmatic randomised controlled trial to compare antidepressants with a community-based psychosocial intervention for the treatment of women with postnatal depression: the RESPOND trial. *Health Technology Assessment* **14**, iii-iv, ix-xi, 1-153.

**Shiovitz, T., Greenberg, W. M., Chen, C., Forero, G. & Gommoll, C. P.** (2014). A Randomized, Double-blind, Placebo-controlled Trial of the Efficacy and Safety of Levomilnacipran ER 40-120mg/day for Prevention of Relapse in Patients with Major Depressive Disorder. *Innovative Clinical Neuroscience* **11**, 10-22.

**Simpson, S., Corney, R., Fitzgerald, P. & Beecham, J.** (2003). A randomized controlled trial to evaluate the effectiveness and cost-effectiveness of psychodynamic counselling for general practice patients with chronic depression. *Psychol ogical Medicine* **33**, 229-39.

**Stewart, J. W., Quitkin, F. M., McGrath, P. J., Rabkin, J. G., Markowitz, J. S., Tricamo, E. & Klein, D. F.** (1988). Social functioning in chronic depression: effect of 6 weeks of antidepressant treatment. *Psychiatry Res* earch**25**, 213-22.

**Swartz, H. A., Frank, E., Zuckoff, A., Cyranowski, J. M., Houck, P. R., Cheng, Y., Fleming, M. A., Grote, N. K., Brent, D. A. & Shear, M. K.** (2008). Brief interpersonal psychotherapy for depressed mothers whose children are receiving psychiatric treatment. *American Journal of Psychiatry* **165**, 1155-62.

**Talbot, N. L., Chaudron, L. H., Ward, E. A., Duberstein, P. R., Conwell, Y., O'Hara, M. W., Tu, X., Lu, N., He, H. & Stuart, S.** (2011). A randomized effectiveness trial of interpersonal psychotherapy for depressed women with sexual abuse histories. *Psychiatric Service*  **62**, 374-80.

**Titov, N., Andrews, G., Davies, M., McIntyre, K., Robinson, E. & Solley, K.** (2010). Internet treatment for depression: a randomized controlled trial comparing clinician vs. technician assistance. *PLoS One* **5**, e10939.

**Trivedi, M. H., Pigotti, T. A., Perera, P., Dillingham, K. E., Carfagno, M. L. & Pitts, C. D.** (2004). Effectiveness of low doses of paroxetine controlled release in the treatment of major depressive disorder. *Journa of Clinical Psychiatry* **65**, 1356-64.

**Unlu Ince, B., Cuijpers, P., van 't Hof, E., van Ballegooijen, W., Christensen, H. & Riper, H.** (2013). Internet-based, culturally sensitive, problem-solving therapy for Turkish migrants with depression: randomized controlled trial. *Journal of Medicine and Internet Research* **15**, e227.

**van Aalderen, J. R., Donders, A. R., Giommi, F., Spinhoven, P., Barendregt, H. P. & Speckens, A. E.** (2012). The efficacy of mindfulness-based cognitive therapy in recurrent depressed patients with and without a current depressive episode: a randomized controlled trial. *Psychological Medicine* **42**, 989-1001.

**van Schaik, A., van Marwijk, H., Ader, H., van Dyck, R., de Haan, M., Penninx, B., van der Kooij, K., van Hout, H. & Beekman, A.** (2006). Interpersonal psychotherapy for elderly patients in primary care. *American Journal of Geriatric Psychiatry* **14**, 777-86.

**Vernmark, K., Lenndin, J., Bjarehed, J., Carlsson, M., Karlsson, J., Oberg, J., Carlbring, P., Eriksson, T. & Andersson, G.** (2010). Internet administered guided self-help versus individualized e-mail therapy: A randomized trial of two versions of CBT for major depression. *Behaioralv Research Therapy* **48**, 368-76.

**Vitriol, V. G., Ballesteros, S. T., Florenzano, R. U., Weil, K. P. & Benadof, D. F.** (2009). Evaluation of an outpatient intervention for women with severe depression and a history of childhood trauma. *Psychiatric Service* **60**, 936-42.

**Ward, E., King, M., Lloyd, M., Bower, P., Sibbald, B., Farrelly, S., Gabbay, M., Tarrier, N. & Addington-Hall, J.** (2000). Randomised controlled trial of non-directive counselling, cognitive-behaviour therapy, and usual general practitioner care for patients with depression. I: clinical effectiveness. *BMJ* **321**, 1383-8.

**Watt, L. M., & Cappeliez, P.** (2000). Integrative and instrumental reminiscence therapies for depression in older adults: Intervention strategies and treatment effectiveness. *Aging & mental health, 4(2), 166-177.*

**Wiles, N., Thomas, L., Abel, A., Ridgway, N., Turner, N., Campbell, J., Garland, A., Hollinghurst, S., Jerrom, B., Kessler, D., Kuyken, W., Morrison, J., Turner, K., Williams, C., Peters, T. & Lewis, G.** (2013). Cognitive behavioural therapy as an adjunct to pharmacotherapy for primary care based patients with treatment resistant depression: results of the CoBalT randomised controlled trial. *Lancet* **381**, 375-84.

**Williams, A. D., Blackwell, S. E., Mackenzie, A., Holmes, E. A. & Andrews, G.** (2013). Combining imagination and reason in the treatment of depression: a randomized controlled trial of internet-based cognitive-bias modification and internet-CBT for depression. *Journal of Consulting and Clinical Psychology* **81**, 793-9.

**Wong, D. F.** (2008). Cognitive and health-related outcomes of group cognitive behavioural treatment for people with depressive symptoms in Hong Kong: randomized wait-list control study. *Australian and New Zealand Journal of Psychiatry* **42**, 702-11.

**Zilcha-Mano, S., Dinger, U., McCarthy, K. S., Barrett, M. S. & Barber, J. P.** (2014). Changes in well-being and quality of life in a randomized trial comparing dynamic psychotherapy and pharmacotherapy for major depressive disorder. *Journal of Affective Disorders* **152-154**, 538-42.

**Zu, S., Xiang, Y. T., Liu, J., Zhang, L., Wang, G., Ma, X., Kilbourne, A. M., Ungvari, G. S., Chiu, H. F., Lai, K. Y., Wong, S. Y., Yu, D. S. & Li, Z. J.** (2014). A comparison of cognitive-behavioral therapy, antidepressants, their combination and standard treatment for Chinese patients with moderate-severe major depressive disorders. *Journal of Affective Disorders* **152-154**, 262-7.
