# Supplementary material for: The efficacy of psychotherapy, pharmacotherapy and their combination on functioning and quality of life in depression: a meta-analysis
Source: Psychol Med. 2016 Oct 26;47(3):414–25. doi: 10.1017/S0033291716002774 (PMC5244449; doi:10.1017/S0033291716002774)
Supplement: Supplementary file 1 [file S0033291716002774sup001.zip › Supplementary material 3.docx]

| 1. (Depressive Disorder [Mesh] OR Major Depressive Disorder [Mesh] OR Depression [Mesh]) |
| --- |
| \| 1. (((((("Disability Evaluation"[Mesh] OR "Sick Leave"[Mesh] OR "Disabled Persons"[Mesh]) OR   "Quality of Life"[Mesh]) OR ( "Activities of Daily Living"[Mesh] OR "Leisure Activities"[Mesh])))  OR "functioning"[Title/Abstract]) \| [507323](http://www.ncbi.nlm.nih.gov/pubmed/?cmd=HistorySearch&querykey=2) \| \| --- \| --- \| |
| 1. "Randomized Controlled Trial" [Publication Type] |
| 1. "Clinical Protocols"[Mesh] |
| 1. (bipolar[Title/Abstract] OR schizoaffective[Title/Abstract]) |
| 1. (#1 AND #2 AND #3) |
| 1. 6 NOT 4 |
| 1. 7 NOT 5 |
| 1. Filters: Humans; English; Adult: 19+ years |

**Search String used in systematic search:**

**PubMed**
