# Supplementary material for: The efficacy of psychotherapy, pharmacotherapy and their combination on functioning and quality of life in depression: a meta-analysis
Source: Psychol Med. 2016 Oct 26;47(3):414–25. doi: 10.1017/S0033291716002774 (PMC5244449; doi:10.1017/S0033291716002774)
Supplement: Supplementary file 1 [file S0033291716002774sup001.zip › Supplementary material 4.docx]

**Multivariate meta-regression analyses**

Var 3 = severity of depression

Var 4 = number of psychotherapeutic sessions

Var 5= sample size

Var 6 = year of publication

Var 7 = duration of treatment

Var 8 = duration of trial

**Psychotherapy against control for functioning**

**Psychotherapy against control for QoL**

**Pharmacotherapy against control for functioning**

**Pharmacotherapy against control for QoL**

**Psychotherapy against pharmacotherapy for functioning**

**Psychotherapy against pharmacotherapy for QoL**

**Combined treatment against pharmacotherapy for functioning**

**Combined treatment against psychotherapy for functioning**

**Combined treatment against psychotherapy and pharmacotherapy for QoL:**

Too few observations
