# Supplementary figures and images for: The efficacy of psychotherapy, pharmacotherapy and their combination on functioning and quality of life in depression: a meta-analysis
Source: Psychol Med. 2016 Oct 26;47(3):414–25. doi: 10.1017/S0033291716002774 (PMC5244449; doi:10.1017/S0033291716002774)

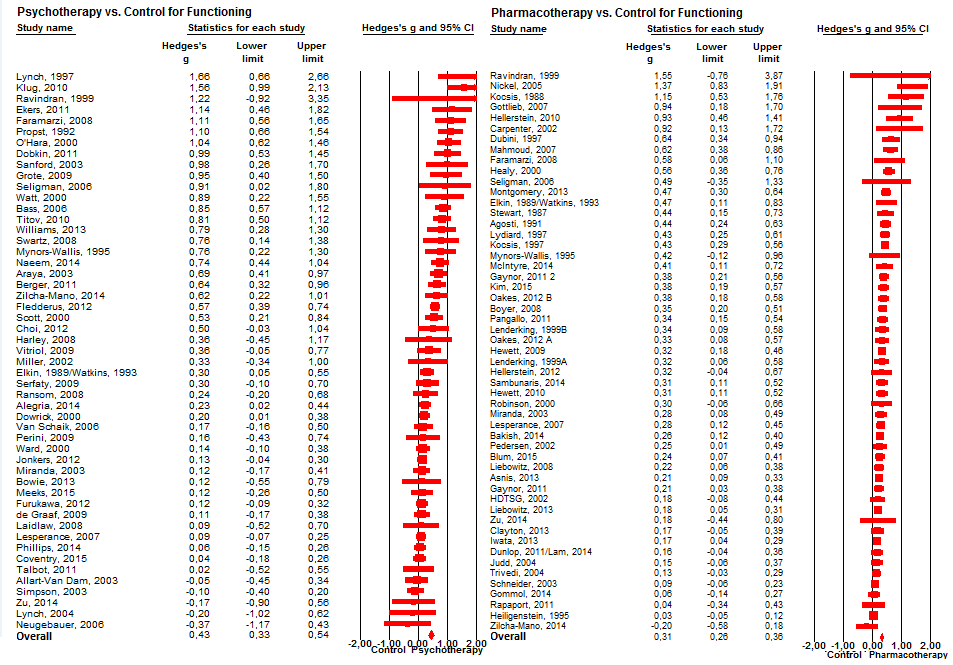

Supplement: Supplementary file 1 [file S0033291716002774sup001.zip › Supplementary material 2A.tif]

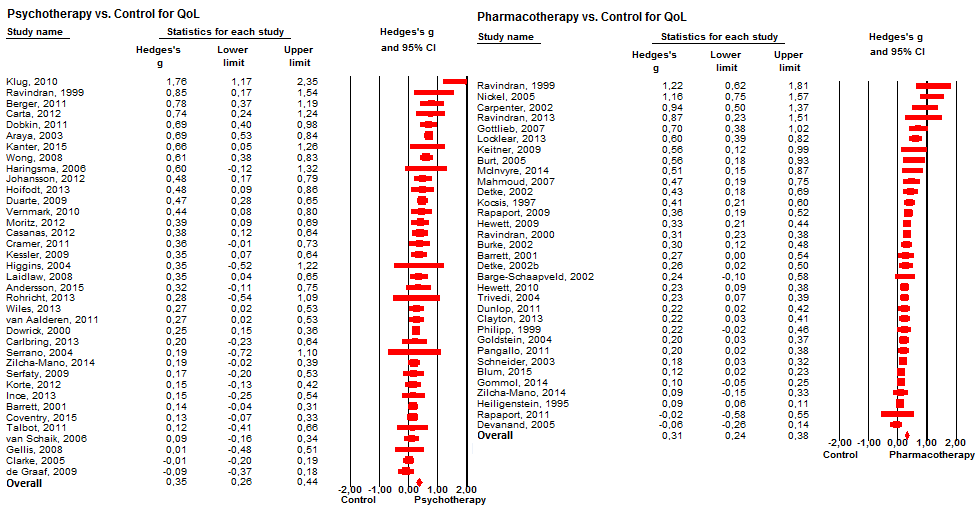

Supplement: Supplementary file 1 [file S0033291716002774sup001.zip › Supplementary material 2B.tif]
